# Morphosyntactic production and processing skills in relation to age effects and lexical-phonological levels among children with cochlear implants and typically hearing peers: a focus on vowel nasality

**DOI:** 10.3389/fnhum.2025.1528388

**Published:** 2025-02-26

**Authors:** Sophie Fagniart, Brigitte Charlier, Véronique Delvaux, Bernard Georges Harmegnies, Anne Huberlant, Myriam Piccaluga, Kathy Huet

**Affiliations:** ^1^Language Sciences and Metrology Unit, University of Mons (UMONS), Mons, Belgium; ^2^Research Institute for Language Science and Technology, University of Mons (UMONS), Mons, Belgium; ^3^Center of Research in Cognition and Neurosciences (CRCN), Université Libre de Bruxelles (ULB), Brussels, Belgium; ^4^Functional Rehabilitation Center “Comprendre et Parler”, Brussels, Belgium; ^5^Fund for Scientific Research-Fonds National de la Recherche Scientifique (F.R.S.–FNRS), Brussels, Belgium

**Keywords:** cochlear implants, morphosyntactic skills, phonological skills, nasal vowels, language development, lexical skills

## Abstract

**Introduction:**

Significant variability in the language performance of children with cochlear implant (CI) is widely recognized in the literature, particularly concerning morphosyntactic (MS) skills. The perceptual limitations of the CI, which can lead to phonological difficulties, may be responsible for this increased vulnerability in grammatical abilities. In this context, the present study focuses on the morphophonemic processing of items distinguished by nasal and oral vowels in the French language – the feature of vowel nasality being known as challenging for the CI population. Links between these performances with chronological/auditory ages and phonological and grammatical production skills will also be explored.

**Method:**

Nineteen children with CIs and 47 children with typical hearing (TH) were assessed for phonological skills through a picture-naming task, perceptual skills through a task involving the sentence/word-picture matching task with word target containing nasal vs. oral vowels, and morphosyntactic production skills through narrative productions. Various measures of linguistic complexity [Mean Length of Utterance (MLU), verbs/utterances (V/U)] and lexical diversity (D index) were evaluated among our groups and linked to perceptual and productive phonological performances. Chronological and auditory ages as well as phonological accuracy and vocabulary levels as been studied as covariates.

**Results:**

Children with CIs displayed significantly lower morphosyntactic (MS) performance compared to peers with TH of the same chronological age, particularly in measures such as MLU in morphemes, complexity of function words, and processing of morphemes carried by nasal and oral vowels. However, when controlling for auditory age or phonological/lexical levels, these differences were no longer significant, suggesting a potential for compensation when similar auditory or linguistic experiences are achieved. Despite this, CI users showed distinct patterns of function word use, with fewer complex forms and more frequent errors, likely reflecting the perceptual challenges linked to CI. Additionally, a specific strong relationship between MS skills and phonological accuracy was observed in the CI group, potentially accounting for the marked inter-individual variability in MS development.

**Conclusion:**

The perceptual limitations of the CI have a significant impact on the linguistic development of children with CI and contribute to the widely documented variability in performance.

## 1 Introduction

The development of language in prelingually deaf children with unilateral or bilateral cochlear implants (CIs) has been the subject of numerous investigations in recent decades. One element is widely agreed upon among researchers: the performance of children with CIs is extremely variable. Some children reach the level of their typically hearing (TH) peers, while others display delayed or even atypical profiles. This variability in performance is not equally distributed across language components: difficulties are most frequently reported in the phonological (Bouton et al., 2012; Nittrouer et al., 2018; David et al., 2021) and morphosyntactic (MS) components (Caselli et al., 2012; Duchesne et al., 2009; Le Normand and Thai-Van, 2023; Rinaldi et al., 2013). Indeed, despite the undeniable benefits of cochlear implantation in auditory perception and in promoting the emergence of spoken language, the CI does not transmit the sound signal with the same precision as typical hearing (TH) (Moon and Hong, 2014). The sound transmitted by the implant is particularly limited in its spectral resolution (Horn et al., 2017; Jahn et al., 2022) and can be imprecise in high- (Reidy et al., 2017; Grandon and Vilain, 2020) and low-frequency ranges (Hochmair et al., 2015; Dincer et al., 2019), impacting the processing of some segmental (Bouton et al., 2012; Romano et al., 2021; Fagniart et al., 2024b), and suprasegmental aspects of speech (Gaudrain and Başkent, 2018; Richter and Chatterjee, 2021; Chatterjee et al., 2023; Frosolini et al., 2023). Furthermore, the period of auditory deprivation before implantation can lead to a lack of stimulation of the auditory pathways during sensitive periods of the development of auditory brain areas (Kral et al., 2016; Karltorp et al., 2020; Sharma et al., 2020). These factors can affect certain language components more severely. The aim of the present study is to examine the MS skills of French-speaking children with CIs compared to their TH peers, as well as the relationships between these skill levels and their lexical and phonological development. Furthermore, this study will also focus on vowel nasality, a distinctive feature of the French phonological system that supports several grammatical markers and has been recognized in the literature as being vulnerable to perception difficulties in both adults and children with CIs (Borel et al., 2019; Fagniart et al., 2024a,^2025^). The ability to process grammatical markers and lexical minimal pairs, whose morphophonological opposition is carried by nasal vowels, will thus be investigated and linked to MS production skills.

Despite the undeniable benefits of cochlear implantation on the linguistic development of deaf children, MS development remains an area of language subject to persistent difficulties in this population. Indeed, lower performances are reported in formal tests evaluating perceptive and productive skills (Young and Killen, 2002; Schorr et al., 2005; Duchesne et al., 2009; Caselli et al., 2012; Bourdin et al., 2016). Linguistic corpus analyses have shown lower values in linguistic development cues such as MLU (Mean Length of Utterance) and/or difficulties in the use of free and bound grammatical morphemes (Szagun, 2001; Hansson et al., 2017; Nittrouer et al., 2018; Majorano et al., 2024) as well as challenges in verbal morphology (Delcenserie et al., 2024). The emergence of function words in the early development of grammar (2–3 years) does not follow the same course as in typically hearing children, with greater difficulties in producing complex, less salient, unstressed function words such as pronouns (subject, object, and relative), possessive, and modal verbs or prepositions (Le Normand, 2004; Le Normand and Thai-Van, 2023). It is suggested that CI users tend to store lexical representations more than phonological ones (Le Normand and Moreno-Torres, 2014), facilitating the acquisition of content words such as nouns or main verbs over function words (Le Normand, 2004; Le Normand and Thai-Van, 2023). Studies that have jointly examined lexical and morphosyntactic components in the same children with CI have shown disparities in performance levels. Indeed, lexical development often appears to be more equivalent to that of peers with TH (Duchesne et al., 2009; Caselli et al., 2012; Rinaldi et al., 2013), with some authors observing a 1-year gap between lexical and morphosyntactic development (Le Normand and Moreno-Torres, 2014). This gap is suggested to be due to the greater prominence of lexical elements in spoken language compared to grammatical words. Moreover, morphological and syntactic variations are more complex to teach/learn formally than vocabulary words (Hage, 2005).

While these difficulties are evident when children with CIs are studied as a group, the study of individual profiles shows substantial variability. Indeed, some studies report performances equivalent to those of typically hearing peers for about 50% of the children matched by chronological age (Geers et al., 2003), auditory age (Guo and Spencer, 2017), or vocabulary size (Jung and Ertmer, 2018). Different inter-individual variability factors can contribute to a certain degree of variability in performance. The effect of auditory age, defined as the age from exposure to auditory stimulation through a CI, has been regularly tested on linguistic components, with contrasting findings depending on the studies. While various authors observe significantly improved linguistic performances with advancing auditory age (Szagun, 2004; Nicholas and Geers, 2007; Wie, 2010; Caselli et al., 2012; Flipsen and Kangas, 2014), others do not report any influence of auditory age (Duchesne et al., 2009; Rinaldi et al., 2013; Hess et al., 2014). It should be noted that numerous individual characteristics of children can also influence their language outcomes, such as the family environment (e.g., family SES, siblings, etc.; Geers et al., 2009; Szagun and Stumper, 2012; Le Normand and Moreno-Torres, 2014) and the level of linguistic stimulation (e.g., input during early life; Holzinger et al., 2020), quality of input (Arjmandi et al., 2022) or methods of language rehabilitation (Van Bogaert et al., 2023). These individual and environmental characteristics, among others, have been identified as potential sources of variability. However, none can fully explain the disparities in performance or predict whether a child with a CI will achieve outcomes equivalent to those of their hearing peers.

Part of this variability could be attributed to perceptual limitations related to sound encoding by the CIs. Indeed, despite the constant evolution of sound coding devices, CIs are still incapable of completely coding and transmitting the auditory signal, as the fine acoustic details are often too complex to be processed by even the most recent devices (for a description of the sources of degraded performance in speech sound coding via the implant, see Başkent et al., 2016). Moreover, these device limitations are associated with the variability linked to the individual settings of the CI and the adequacy of the MAPping (Lee et al., 2012; Mittal et al., 2015; Kocabay et al., 2022), as well as the daily use of the device (Glaubitz et al., 2021; Wiseman et al., 2021), which can influence children’s auditory experience and language outcomes. This limitation impacts the quality of the phonological representations in prelingually deaf children, which directly affects their phonological skills as well as their overall linguistic skills, particularly their grammatical skills. The increased vulnerability of morphosyntactic skills in cases of perceptual and/or phonological limitations has been explained by various models developed to explain SLI, where these skills are most severely affected (Parisse and Maillart, 2007). For instance, the surface hypothesis (Leonard et al., 1992) suggests that grammatical morphemes are more vulnerable due to their typical final positions and placement in unstressed syllables. The cognitive operation of attributing a grammatical mark is also complex, making these morphemes more likely to be processed imprecisely, impacting grammatical development in both perception and production. Joanisse and Seidenberg (1998) propose a theoretical model that also emphasizes the lack of perceptual salience of morphological markers, adding that the root cause of difficulties lies initially in perceptual issues. This leads to difficulties in perceiving and categorizing phonological contrasts of the language and results in underspecified phonological representations. Since morphosyntactic markers primarily rely on contrasts between morphophonological forms, they are likely to be problematic in this context. The mapping hypothesis (Chiat, 2001) complements these propositions by postulating that developmental language disorders are related to a deficit in mapping between a phonological form and its concept/referent, which can then affect lexical and morphosyntactic development. However, lexical and morphosyntactic items do not hold the same value in terms of conceptual representation: concepts associated with lexical item are more frequently linked to visual, social, or emotional cues, unlike grammatical marks which rely solely on phonological cues from the spoken chain. In the case of children with hearing loss, the limitation and/or degradation of auditory input, causing a perceptual deficit, will be even more pronounced on less salient and more abstract elements of the language, such as grammatical morphemes. This could explain why the morphosyntactic level is most often deficient compared to the lexical level in this population (Caselli et al., 2012; Duchesne et al., 2009; Le Normand and Moreno-Torres, 2014; Rinaldi et al., 2013), with lexical items benefiting from greater salience and concreteness effects. This could be corroborated by the study of Hansson et al. (2017) which found a strong link between the level of phonological development, assessed through a non-word repetition task, and skills in sentence comprehension as well as grammatical accuracy, evaluated through narrative production in children with CIs aged 5–9 years. The authors attributed these difficulties to imprecise phonological representations due to degraded auditory input, impacting the processing of grammatical morphemes both in processing and morphosyntactic production.

The difficulties in the processing of speech sounds by CI users affect more severely certain phonological features carried by acoustic cues less precisely coded by the CI (Bouton et al., 2012; Cheng et al., 2021; Moon and Hong, 2014; Peng et al., 2019). This is particularly the case for the nasality feature in French vowels. In French, as in approximately 20% of the world’s languages (Borel, 2015), nasality is a distinctive feature of the vocalic system. The [nasal] vs. [oral] specification allows for the distinction of minimal pairs at the lexical level, but also morphophonological oppositions that serve as grammatical markers, such as grammatical number in /il va/ (“he goes”) vs. /il vÞ/ (“they go”). However, vowel nasality is carried by fine acoustic cues that require optimal spectral resolution processing skills, which is precisely problematic in the processing of the sound signal by the CI. Various experimental studies have thus shown difficulties in the identification and discrimination of nasal and oral vowels in both children and adults with CI(s) (Bouton et al., 2012; Borel, 2015; Borel et al., 2019; Fagniart et al., 2024a). CI users tend to have difficulty distinguishing a nasal vowel from a close oral counterpart in terms of oropharyngeal configuration. Their perceptual difficulties, mainly concerning the processing of the nasal quality of the vowel, so that the identification/discrimination of nasal and oral vowels is primarily carried out through the exploitation of cues better coded by the CI, such as formant frequencies. These perceptual difficulties manifest in specific productive profiles. Indeed, children with CIs judged to be the most intelligible are those who distinguish nasal and oral vowels using acoustic cues related to oropharyngeal configuration (formant frequencies) rather than presence or absence of nasal resonance (Fagniart et al., 2025).

In this context, we have chosen to study morphosyntactic production skills as well as the processing skills of different morphemic oppositions in grammatical and lexical contexts among groups of children with CIs and their TH peers. Morphosyntactic production will be studied through narrative production tasks, allowing the collection of various developmental cues, the inventorying of function word types, verb moods, and tenses produced, as well as the errors made by the children. Additionally, a customized comprehension task designed to study morphophonological processing will be introduced. The inclusion of different types of morphophonological oppositions, some of which based on vowel nasality, will allow us to assess the impact of perceptual difficulties on morphological processing skills in various grammatical (e.g., gender and number marking) and lexical (e.g., minimal pairs) contexts. Morphemic oppositions involving vowel nasality have been targeted because, as indicated in previous literature (Borel et al., 2019; Fagniart et al., 2024a,^2025^), the nasal vowel feature is poorly perceived among the CI population, leading to difficulties in discrimination and peculiarities in production, but with possible compensation strategies.

Furthermore, given the significant inter-individual variability reported in the literature regarding the linguistic development of TH children, and even more so in populations with CI(s), it was decided to conduct various types of comparisons. Indeed, comparisons based on chronological age between TH and CI groups is the most common comparison used in literature. However, this type of comparison does not account for the period of auditory deprivation before implantation, which may have delayed access to full auditory input. Comparisons based on auditory age may be relevant to address this, enabling the comparison of children with more similar auditory experiences (Duchesne et al., 2009; Caselli et al., 2012). However, this type of comparison involves comparing CI children to younger TH children, who therefore have a less mature cognitive level, which may also affect the comparability of results (Faes and Gillis, 2016; Jung and Ertmer, 2018). In this regard, various authors recommend including comparisons based on linguistic level, through comparisons in terms of vocabulary size (Duchesne et al., 2009; Faes and Gillis, 2016; Jung and Ertmer, 2018). In this study, these different types of comparisons will be included, allowing the positioning of TH and CI groups relative to their chronological age, auditory experience (auditory age), and linguistic level (vocabulary size and phonological precision). Although vocabulary and phonological development levels have been deemed commensurate in children (Stoel-Gammon, 2011; Sosa and Stoel-Gammon, 2012), these two variables will be measured separately in this study to specifically assess their respective roles in MS development. Indeed, if phonological theories of MS development explain specific difficulties in children with CI, a greater involvement of phonological development compared to lexical development might be observed. In this sense, comparisons will be made between these covariates to identify which ones best predict the data. Finally, the various scores obtained from this comprehension task will also be linked to different morphosyntactic production scores obtained through narrative production, to study the connections between potential morphological processing difficulties and grammatical skills within the two groups of children.

Several objectives are pursued:

1. Measures of phonological and lexical development are collected and compared between the TH and CI groups to use them as covariates in group comparisons. Greater difficulties are expected in the phonological domain than in the lexical domain among CI children, as the latter is more frequently reported in the literature as relatively preserved (Duchesne et al., 2009; Caselli et al., 2012; Rinaldi et al., 2013), whereas phonology is often identified as the most affected component (Nittrouer et al., 2014, 2018).
2. Grammatical production skills are documented through narrative analysis. Similar studies conducted in French with younger children (Le Normand, 2004; Le Normand and Thai-Van, 2023) have highlighted an atypical acquisition trajectory of function words among young CI users compared to children with TH. The focus of the present study is on older age groups to document performance in morphosyntactic production, including the production of various function words, verb moods, and tenses, as well as different types of grammatical errors among the groups of children.
3. Morphological processing skills in grammatical and lexical contexts are documented. Greater difficulties are hypothesized for syntagms distinguished by nasal vowel features among CI users.
4. The role of different variables contributing to morphosyntactic development and the variability in performance observed among CI users is compared. Maturation effects reported in the literature suggest more comparable language performance when auditory experience is accounted for by comparing auditory ages (Caselli et al., 2012; Guo and Spencer, 2017). Group effects are analyzed by considering both chronological and auditory ages. Phonological and lexical developmental levels are also used to control group effects. This approach examines whether performance differences persist at equivalent lexical or phonological levels while assessing the predictive value of these linguistic levels on performance. A stronger predictive value of phonological developmental levels could provide support for phonological theories of morphosyntactic development disorders (Leonard et al., 1992; Joanisse and Seidenberg, 1998; Chiat, 2001).
5. Finally, links between productive skills and morphemic processing skills, particularly in the context of morphophonological opposition between nasal and oral vowels, are studied to determine whether the processing of morphemes conveyed by fine spectral cues is associated with better performance in MS production.

## 2 Materials and methods

### 2.1 Participants

A group of children with typical hearing (TH group) and a group of children with cochlear implants (CI group) participated in the study. The TH group consisted of 47 French-speaking children with an average age of 56 ± 7 months, who did not exhibit any learning delays or auditory disorders. The CI group consisted of 19 French-speaking children (mean chronological age: 64 ± 2 months) with congenital bilateral profound hearing loss. Among them, 18 had bilateral implants, and 1 child had a unilateral implant without a contralateral hearing aid, with an average age of first implantation of 17.2 ± 8.7 months. All CI participants received oralist auditory rehabilitation, both at their rehabilitation center and in their family environment. Approximately half (11/19) of the children were also exposed to sign language in addition to spoken language, although the preferred mode of communication remained oral. The list of CI participants and their characteristics is presented in Table 1.

**TABLE 1:** Characteristics of the children with CIs.

Subject	Sex	Chronological age (years; months)	Age at first implantation (months)	Implantation type
CI1	M	4;6	9	Bilateral
CI2	M	6;5	39	Bilateral
CI3	F	5;10	15	Bilateral
CI4	F	6;7	7	Bilateral
CI5	F	6;6	31	Bilateral
CI6	F	4;7	7	Unilateral
CI7	F	7;3	13	Bilateral
CI8	M	4;7	13	Bilateral
CI9	M	4;9	13	Bilateral
CI10	M	4;6	12	Bilateral
CI11	F	4;6	18	Bilateral
CI12	F	6;9	20	Bilateral
CI13	M	6;0	23	Bilateral
CI14	F	3;9	12	Bilateral
CI15	F	5;0	32	Bilateral
CI16	F	3;8	11	Bilateral
CI17	M	4;11	17	Bilateral
CI18	F	6;7	13	Bilateral
CI19	F	5;0	21	Bilateral

### 2.2 Tasks

#### 2.2.1 Naming task

The children first completed a picture-naming task. The target words (*n* = 48) for this task were selected by the authors to include all French phonemes in initial, medial, and final syllable positions. The target words were also chosen for their early age of acquisition (referring to Chalard et al., 2003) to facilitate retrieval by the children (for more information, see Philippart De Foy et al., 2018).

The target word pictures were presented to the child one at a time via a booklet, and the child was asked to orally name each picture. If the child did not respond or if the produced word did not match the target (e.g., semantic paraphasia or a random response), different prompts were provided. First, semantic cues related to the target word were given (e.g., “you can use it when it rains” for /paʁaplɥi/ – *umbrella*). If the target word was still not produced, a phonological cue was offered by providing the initial phoneme (e.g., “it starts with /s/” for /suri/ – *mouse*). If these two cues were insufficient for the child to retrieve the target word, the experimenter would say the word and ask the child to repeat it.

#### 2.2.2 Narrative production tasks

Two narrative production tasks were proposed to the children: an induced narrative task and a free narrative task.

The first narrative task was the induced narrative. In the initial phase, a story with images was presented to the children. An animated story was shown on a tablet, with the animations illustrating the story to provide visual support, while a filmed narrator told the story. In order to prevent the child from focusing solely on the narrator or the animations, thereby missing information, the phases of narration by the speaker and the animation phases alternated without overlapping, allowing the child to shift from one to the other. Afterward, the child was asked to retell the story using the animations previously shown as visual support. The purpose of the induction phase was to present a story containing various twists and turns that would introduce past, future, and conditional tenses/modes, as well as gender and number markers. The goal was to encourage varied productions of grammatical markers and verb tenses from the children.

The free narrative task involved presenting the wordless picture book “Frog, Where Are You?” (Mayer, 1969). The child was shown the book and asked to tell the story.

#### 2.2.3 Sentence/word-picture matching task scores

The comprehension task consisted in a sentence/word-picture matching task. A word or a short sentence was presented auditorily to the children, and they were asked to point to the corresponding picture in a pair of images. The task included a total of 28 items. The differences between the target words/sentences and their distractors involved : 13 number markings [e.g., “il va” – /il va/ (*he goes*) vs. “ils vont” – /il v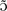/ (*they go*)], 7 gender markings [e.g., “boulanger” – /bul
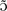
ʒe/ (*baker – male*) vs. “boulangère” – /bulãʒε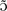/ (*baker – female*)] and 8 lexical minimal pairs [e.g., “bain” – /b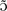/ (*bath*) vs. “banc” – /bã/ (*bench*)]. These different grammatical and lexical distinctions were based on various phonological contrasts: oral/nasal (*n* = 10), oral/oral (*n* = 3), or nasal/nasal (*n* = 3) vowel substitutions, as well as phonemic additions (*n* = 12).

The children were presented with two images (the target image and the distractor) on a tablet. They listened to the target word or sentence through an audio recording played via loudspeakers (Bose Soundlike). The sound level was controlled to reach an average level of 60 dB. The children were then asked to point to the corresponding target image among the two images. Five practice items were provided before the task to ensure the children understood the instructions and to adjust the sound volume for optimal listening of the stimuli.

### 2.3 Procedure

The children completed the four tasks in a quiet environment, in the presence of the experimenter and, in some cases, their speech therapist. The tasks were administered in the following order: first, the picture-naming task, followed by the induced narrative production task, the comprehension task, and finally, the free narrative production task. The total testing time ranged between 35 and 60 min. Breaks were proposed to the children between tasks. All of the children’s productions were recorded using an H5 Zoom portable audio recorder.

### 2.4 Measures

#### 2.4.1 Phonological score

The children’s productions in the naming task were annotated by an initial examiner and subsequently verified by the first author using the Phon 3.1 software (Hedlung and Rose, 2020). The software, by comparing the target phonological form with the actual production as annotated through a narrow transcription, was able to extract various phonological accuracy scores. In this study, we will focus solely on a global score of percentage of correct phonemes. A more comprehensive description of the children’s phonological as well as acoustic analyses of the productions are available in a previous study (Fagniart et al., 2024a).

#### 2.4.2 Grammatical production scores and lexical score

The children’s productions from the audio recordings of the narratives were transcribed using PHON (Hedlung and Rose, 2020) and then exported to the Computerized Language Analysis (CLAN) software (MacWhinney, 2000) for the purpose of conducting a morphosyntactic annotation of the words in the narratives using the MOR and POST functions (Parisse and Le Normand, 2000). The KidEval program was used to extract various cues of morphosyntactic development, while also classifying the different function words produced and the verb tenses used.

For the study, we focused on the following cues for analysis, known to be indicators of morphosyntactic development or linked to morphosyntactic complexity:

1. Mean Length of Utterance (MLU) in morphemes (MLUm)
2. The verb/utterance ratio: determining the number of utterances containing a main verb
3. The number of the following function and content words: prepositions, pronouns, demonstrative pronouns, reflexive pronouns, object pronouns, subject pronouns, adjectives, adverbs, articles, possessive determiners, conjunctions, number of regular verbs, copula verbs, modal verbs, auxiliary verbs, and possessive verbs. The raw counts of these different grammatical words were divided by the total number of words produced by the child, in order to obtain a relative measure that is not influenced by sample size.
4. Verb forms related to variations in tenses and moods: present, past simple (judged as equivalent to the “imparfait” in French), past perfect (judged as equivalent to the “participe passé” in French), conditional, and future. The raw counts of these different verb forms were divided by the total number of verbs produced by the child.

The annotated narratives were also reviewed by the first author to identify different types of errors, which will be analyzed. The errors observed included:

– Noun agreement errors in number [“*les cheval*” – /le∫əval/ – i.e., “*the (plural) horse (singular)*” instead of “*les chevaux*” – /le∫əvo/ – i.e., “*the (plural) horses (plural*)”] and gender [“*la boulanger*” – /labulãʒe/ – i.e., “*the baker (male)*” instead of “*la boulangère*” – /labulãəɛʁ/ – i.e., “*the baker (female)*”]
– Verb agreement errors in number (“*les amis vient*” – /lezamivj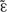/ – i.e., “*the friends is coming*” instead of “*les amis viennent*” – /lezamivjεn/ – i.e., “*the friends are coming*”)
– Verb form errors: auxiliary (“*il a parti*” – /ilapaʁti/ instead of “*il est parti*” – /ilεpaʁti/), form (“*ils se marier*” – /ilsəmaʁje/ instead of “*ils se mariaient*” – /ilsəmaʁjε/), overgeneralization (“*il parta*” – /ilpaʁta/ instead of “*il partit*” – /ilpaʁti/)
– Substitution of function words: preposition (“*il tombe sur la fenêtre*” – /ilt
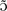
bsyʁlafənϵtʁ/ instead of “*il tombe par la fenêtre*” – /ilt
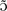
bpaʁlafənϵtʁ/), contracted article (“*de le cheval*” – /dələ∫əval/, instead of “*du cheval*” – /dy∫əval/), clitic pronoun (“*il le regarde (mention to a female character*” – /illəʁəgaʁd/, instead of “*il la regarde*” – /illaʁəgaʁd/), others
– Deletion of function words: determiners (“*chien*” – /∫əval/, instead of “*le chien”* – /lə∫j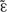/), prepositions (“il *tombe la fenêtre*” – /il t
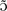
b la fənεt/ instead of “*il tombe par la fenêtre*” – /ilt
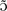
bpaʁlafənεtʁ/)
– Addition of function words (“*il vend en ses produits*” – /il vãã sε pʁodyi/ instead of “*il vend ses produits*” – /il vã sε pʁodyi/)

In order to also obtain an indicator related to the level of lexical development, the lexical diversity index D, derived from the VOCD procedure in KidEval (Duran et al., 2004), was computed. This index was created to avoid being influenced by sample size, unlike the Type/Token Ratio (TTR) index, which increases with the size of the corpus. The index is calculated based on a mathematical model of the probability that a new word will be introduced as the corpus lengthens. This mathematical model is compared to the actual produced corpus to obtain the D index. This index has proven to be more reliable for evaluating corpora of different sizes and has demonstrated its ability to discriminate between different types of speakers (Duran et al., 2004).

All these measures were conducted on both narratives (induced and free), analyzed jointly.

#### 2.4.3 Sentence/word-picture matching task scores

For the comprehension task, *d*′ scores were calculated for all scores related to a specific category. First, scores were computed for each type of grammatical marker involved in the comprehension task (gender and number), as well as for all the items including minimal pairs. Second, specific *d*′ scores were calculated for each of the phonological contrasts conveying the distinction between the target and the distractor: contrasts between nasal and oral vowels, oral and oral vowels, nasal and nasal vowels, or the phonological process of phonemic addition. *d*′ scores were obtained by subtracting the normalized, centered, and standardized values of hit (correct detection) and false alarm (incorrect detection) rates, according to the signal detection theory (MacMillan and Creelman, 1991). Extreme scores of 0 and 1 were converted to 0.01 and 0.99, respectively, to allow for *Z*-score conversion.

### 2.5 Statistical analysis

Linear generalized mixed models, implemented using the lme4 package (version 1.1-34; Bates et al., 2015) within the R software environment (R Core Team, 2022), were used to compare groups across various measures of the children’s speech productions. The models included two fixed effects: the variable classifying children according to their auditory status (CI vs. TH) and one of the covariates under study, namely chronological age, auditory age (as measured by the age from the first implantation in the CI group), vocabulary level (as measured by the lexical diversity index D from narrative productions), or phonological accuracy (percentage of correct phonemes obtained in the picture-naming task). Interactions between auditory status and each of the covariates were also tested. A random intercept effect for each subject was included in the models. The statistical significance of fixed effects for auditory status was assessed using *t* values and *p* values derived from the model estimates. For interaction effects, Chi-square statistics and *p* values were calculated using the ANOVA function from the car package (Fox and Weisberg, 2018). Model comparisons were performed using the Akaike information criterion (AIC) to identify the best predictor of performance. Pearson correlation coefficients were calculated between the various measures.

## 3 Results

### 3.1 Vocabulary and phonological accuracy level

**FIGURE 1:**
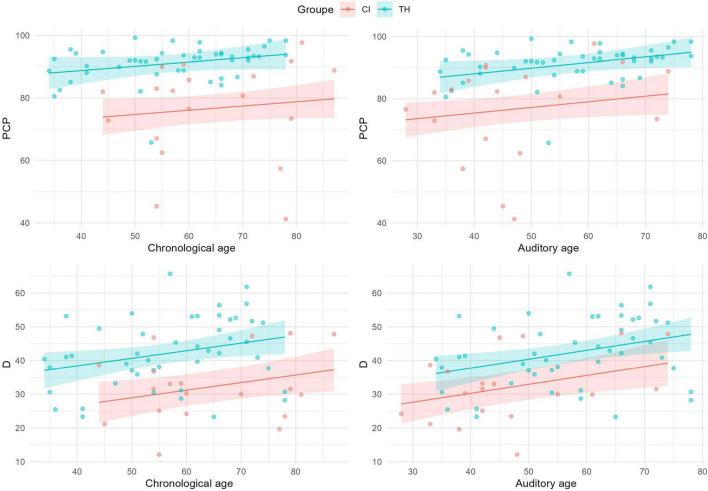
Scatterplots of percentage scores of correct phonemes (PCP – **top**) and lexical diversity index (D – **bottom**) as a function of chronological age **(left)** and auditory age **(right)** in months, for CI (red) and TH (blue) groups. Regression lines with 95% prediction intervals, based on the tested mixed models, are included.

Vocabulary and phonological precision levels were assessed respectively through lexical diversity scores during the production of two narratives and the percentages of correct phonemes in a picture-naming task. Figure 1 illustrates these measures as a function of chronological and auditory age across the CI and TH groups. When controlling for chronological age as a covariate, higher scores were observed in the TH group for both lexical diversity scores (CI: 31.0; TH: 42.7; β = 11.7, SD = 2.8, *t*(63) = 4.2, *p* < 0.001) and percentages of correct phonemes (CI: 76.0; TH: 91.4; β = 15.4, SD = 2.7, *t*(63) = 5.7, *p* < 0.001). Using auditory age as a covariate yielded similar results: while children in the CI group demonstrated higher scores compared to their chronological age, their performance remained significantly lower than the TH group for both lexical diversity scores (CI: 34.0; TH: 41.5; β = 7.5, SD = 2.8, *t*(63) = 2.6, *p* = 0.01) and percentages of correct phonemes (CI: 78.0; TH: 90.6; β = 12.6, SD = 2.7, *t*(63) = 4.6, *p* < 0.001). Notably, significant variability was observed in the performance of children in both groups, as illustrated by the number of data points falling outside the confidence intervals of the regression lines, particularly for the percentage of correct phonemes in the CI group (auditory age: 31.6% of subjects below the confidence interval vs. 19.1% in the TH group). A larger difference can be observed between the two groups in the percentages of correct phonemes compared to lexical diversity scores.

### 3.2 Results of narrative productions

#### 3.2.1 Developmental cues

**FIGURE 2:**
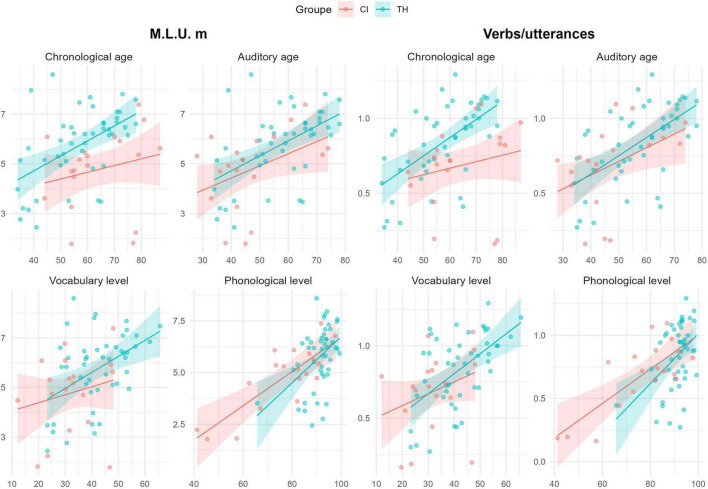
Scatterplots of percentage scores for Mean Length of Utterances in morphemes (MLUm – **left panels**) and the verbs/utterances ratio **(right panels)** as a function of chronological age **(top left)**, auditory age **(top right)** in months, vocabulary **(bottom left)**, and phonological level **(bottom right)** for CI (red) and TH (blue) groups. Regression lines with 95% prediction intervals, based on the tested mixed models, are included.

Figure 2 presents the mean values of MLUm and the V/U ratio as a function of chronological and auditory ages, as well as vocabulary and phonological levels, across the CI and TH groups. Higher values are observed for MLUm (CI: 4.52; TH: 5.86; β = 1.33, SD = 0.38, *t*(63) = 3.4, *p* = 0.001) and the V/U ratios (CI: 0.64; TH: 0.86; β = 0.22, SD = 0.06, *t*(63) = 3.4, *p* = 0.001) in the TH group, with a faster progression evident when data is analyzed as a function of chronological age. However, these group differences disappear when the data are analyzed as a function of auditory age, vocabulary levels, or phonological precision. Significant effects of all covariates are observed for both indices: chronological age (MLUm: β = 0.05, SD = 0.01, *t*(63) = 3.7, *p* < 0.001; V/U ratio: β = 0.009, SD = 0.002, *t*(63) = 4.4, *p* < 0.001), auditory age (MLUm: β = 0.05, SD = 0.01, *t*(63) = 4.4, *p* < 0.001; V/U ratio: β = 0.01, SD = 0.002, *t*(63) = 5.2, *p* < 0.001), vocabulary level (MLUm: β = 0.05, SD = 0.02, *t*(63) = 3.2, *p* = 0.001; V/U ratio: β = 0.01, SD = 0.002, *t*(63) = 4.3, *p* < 0.001), and phonological precision (MLUm: β = 0.09, SD = 0.02, *t*(63) = 5.6, *p* < 0.001; V/U ratio: β = 0.02, SD = 0.003, *t*(63) = 5.3, *p* < 0.001). Notably, models that include phonological precision as a covariate show the lowest AIC values, suggesting that phonological precision is a key factor in explaining variations in MLUm and V/U ratios.

#### 3.2.2 Function words

Table 2 displays the significance of the tests for group and covariate effects across the different covariates related to the function word of interest, as well as the best covariate model based on AIC values. The figures representing these values as a function of the different covariates, by group and by function word, are available in Supplementary Appendix 1.

**TABLE 2:** Summary of group effects (CI vs. TH), target covariate effects (chronological age – CA, auditory age – AA, vocabulary level – VL, and phonological level – PL), and their interactions for the various function words studied.

	Chronological age comparison			Auditory age comparison			Vocabulary level (VL) comparison			Phonological level (PL) comparison			Best model
	Group effect	CA effect	Group × CA	Group effect	AA effect	Group × AA	Group effect	VL effect	Group × VL	Group effect	PL effect	Group × PL	
Adjective	*	*	*					*		*	*	**	VL
Adverb		*			*			***			**		VL
Conjunction	*	*		**			*	*		*			CA
Article determiner							*	***					VL
Possessive determiner	***			*			*	*		*			VL
Preposition	***			**	*		*	**		**	***	*	PL
Demonstrative pronoun		***			***								AA
Object pronoun	*						*	**	*				VL
Reflexive pronoun	***			*	*		*	**		*			VL
Subject pronoun			*		*						**		PL

Significant effects are indicated with *p < 0.05, **p < 0.005, or ***p < 0.001. The model with the lowest AIC (best model) is listed on the right.

Different trends between the groups are observed depending on the function words studied. Some function words, such as possessive determiners, are observed with greater prevalence in the TH group regardless of the covariate type (chronological age: β = 0.88, SD = 0.27, *t*(63) = 3.3, *p* = 0.001; auditory age: β = 0.72, SD = 0.28, *t*(63) = 2.6, *p* = 0.01; vocabulary level: β = 0.58, SD = 0.28, *t*(63) = 2.1, *p* = 0.04; and phonological level: β = 0.87, SD = 0.32, *t*(63) = 2.7, *p* = 0.008). Similar patterns are found for prepositions (chronological age: β = 2.83, SD = 0.69, *t*(63) = 4.1, *p* < 0.001; auditory age: β = 2.2, SD = 0.71, *t*(63) = 3.1, *p* = 0.002; vocabulary level: β = 1.76, SD = 0.7, *t*(63) = 2.5, *p* = 0.01; and phonological level: β = 1.55, SD = 5.8, *t*(63) = 3.1, *p* = 0.003) and reflexive pronouns (chronological age: β = 0.78, SD = 0.2, *t*(63) = 3.7, *p* < 0.001; auditory age: β = 0.5, SD = 0.2, *t*(63) = 2.4, *p* = 0.01; vocabulary level: β = 0.43, SD = 0.2, *t*(63) = 1.99, *p* = 0.05; and phonological level: β = 0.63, SD = 0.2, *t*(63) = 2.5, *p* = 0.01). Articles are also observed in higher proportions in the TH group when vocabulary level is used as a covariate (β = 2.4, SD = 0.95, *t*(63) = 2.5, *p* = 0.01), as are object pronouns when the data are analyzed as a function of chronological age (β = 0.3, SD = 0.1, *t*(63) = 2.6, *p* = 0.01) and vocabulary level (β = −0.72, SD = 0.34, *t*(63) = −2.1, *p* = 0.04). Adjectives, on the other hand, show significant group differences when chronological age (β = 2.99, SD = 1.4, *t*(63) = 2.2, *p* = 0.03) and phonological level (β = −6.7, SD = 2.5, *t*(63) = −2.6, *p* = 0.01) are used as covariates. Conversely, conjunctions are observed in greater numbers in the CI group (chronological age: β = −1.17, SD = 0.57, *t*(63) = −2, *p* = 0.04; auditory age: β = −1.6, SD = 0.6, *t*(63) = −2.7, *p* = 0.009; vocabulary level: β = −4.2, SD = 2, *t*(63) = −2, *p* = 0.04; and phonological level: β = −1.7, SD = 0.7, *t*(63) = −2.5, *p* = 0.01).

An interaction effect is observed for adjectives with chronological age (χ^2^(1) = 4.78, *p* = 0.03) and phonological level (χ^2^(1) = 7.5, *p* = 0.005) as covariates. Specifically, values increase with chronological age in the CI group but decrease slightly in the TH group, while the opposite trend is observed for phonological level. Similarly, an interaction effect is noted for subject pronouns between the group and the covariates chronological age (χ^2^(1) = 4, *p* = 0.04): CI children’s values decrease with age, whereas those of TH children increase. Further interaction effects include object pronouns (χ^2^(1) = 5.9, *p* = 0.01), where TH children’s values increase with vocabulary level while CI children remain stable. Finally, the prevalence of adverbs shows no significant association with group effects, regardless of the covariate.

#### 3.2.3 Verbal morphology

Table 3 displays the significance of the tests for group and covariate effects across the different covariates related to the various verbal forms observed in the narrative tasks, as well as the best covariate model based on AIC values. Figures representing these values as a function of the different covariates, by group and by function word, are available in Supplementary Appendix 2.

**TABLE 3:** Summary of group effects (CI vs. TH), target covariate effects (chronological age – CA, auditory age – AA, vocabulary level – VL, and phonological level – PL), and their interactions for the various verb moods and tenses studied.

	Chronological age comparison			Auditory age comparison			Vocabulary level (VL) comparison			Phonological level (PL) comparison			Best model
	Group effect	CA effect	Group × CA	Group effect	AA effect	Group × AA	Group effect	VL effect	Group × VL	Group effect	PL effect	Group × PL	
Present	***	***			***			***		***	*		CA
Future	*	*			*			*					VL
Conditional	**	*			*			***					VL
Past simple	***	***			***			**					AA
Subjunctive											*		PL
Past perfect	**			**				***	**		*		VL
Infinitive		*											CA

Significant effects are indicated with *p < 0.05, **p < 0.005, or ***p < 0.001. The model with the lowest AIC (best model) is listed on the right.

With chronological age as a covariate, a greater number of indicative future (β = 0.91, SD = 0.35, *t*(63) = 2.6, *p* = 0.01), conditional (β = 1.7, SD = 0.6, *t*(63) = 2.8, *p* = 0.007), and past simple (β = 10.9, SD = 2.9, *t*(63) = 3.7, *p* < 0.001) forms are observed in the TH group. However, this effect is not observed when auditory age, vocabulary, or phonological levels are used as covariates. The use of the indicative present is observed in greater numbers in the CI group when chronological age is used as a covariate, but no group differences are found when auditory age, vocabulary, or phonological levels are considered. Regarding the past perfect, a greater prevalence is observed in the CI group across all covariates (chronological age: β = −9.76, SD = 3.3, *t*(63) = −3, *p* = 0.004; auditory age: β = −10.4, SD = 3.4, *t*(63) = −3.1, *p* = 0.003; vocabulary level: β = −10.9, SD = 3.5, *t*(63) = −3.6, *p* < 0.001; and phonological level: β = −8.9, SD = 3.4, *t*(63) = −1.96, *p* = 0.05), along with an interaction effect with vocabulary (χ^2^(1) = 10.7, *p* = 0.001). Specifically, the prevalence of the past perfect decreases as D index values increase.

#### 3.2.4 Errors

Table 4 displays the significance of the tests for group and covariate effects across the different covariates related to the various error types observed in the narrative tasks, as well as the best covariate model based on AIC values. Figures representing these values as a function of the different covariates, by group and by function word, are available in Supplementary Appendix 3.

**TABLE 4:** Summary of group effects (CI vs. TH), target covariate effects (chronological age – CA, auditory age – AA, vocabulary level – VL, and phonological level – PL), and their interactions for the various grammatical errors studied.

		Chronological age comparison			Auditory age comparison			Vocabulary level (VL) comparison			Phonological level (PL) comparison			Best model
		Gr	CA	Gr × CA	Gr	AA	Gr × AA	Gr	VL	Gr × VL	Gr	PL	Gr × PL	
Nominal agreement	Number													PL
	Gender	0.08			0.06									PL
	i.e., determiner + noun	**			**			**				*		PL
Verbal agreement	Number				*									AA
Verbal tense	Auxiliary	**	**	**	*	*	*	*	*		**		**	PL
	Over-generalization								*					VL
	Form	*						*	*	*		*		VL
Omission	Determiners	***			**			**				***		PL
	Preposition	***			**			**			***			AA
	Pronoun	*							*			***		PL
Addition		*			*	*		*			*			PL

Significant effects are indicated with *p < 0.05, **p < 0.005, or ***p < 0.001. The model with the lowest AIC (best model) is listed on the right.

No significant group differences were observed for nominal agreement in number. However, for nominal agreement in gender, CI children made more errors, approaching significance with chronological age (β = −0.18, SD = 0.2, *t*(63) = −1.7, *p* = 0.08) and auditory age (β = −0.2, SD = 0.1, *t*(63) = −1.85, *p* = 0.06) as covariates. When focusing specifically on errors within determiner-noun syntagms, significantly more errors were observed in the CI group across three covariates: chronological age (β = −0.28, SD = 0.1, *t*(63) = −3.2, *p* = 0.002), auditory age (β = −0.25, SD = 0.1, *t*(63) = −2.8, *p* = 0.006), and vocabulary level (β = −0.25, SD = 0.1, *t*(63) = −2.7, *p* = 0.008). For verbal agreement in number, CI children made significantly more errors when auditory age was used as a covariate (β = −0.08, SD = 0.04, *t*(63) = −2.3, *p* = 0.02).

A greater number of verb morphology errors was also observed in the CI group. Regarding verb tense errors, children in the CI group showed a higher frequency of auxiliary misuse across all the covariates: chronological age (β = −0.45, SD = 0.1, *t*(63) = −3.1, *p* = 0.002), auditory age (β = −0.3, SD = 0.2, *t*(63) = −2.6, *p* = 0.01), vocabulary level (β = −0.22, SD = 0.1, *t*(63) = −2.1, *p* = 0.04), and phonological level (β = −0.82, SD = 0.27, *t*(63) = −3.1, *p* = 0.003). Interaction effects between group and covariates were found for chronological age (χ^2^(1) = 8.3, *p* = 0.003) and auditory age (χ^2^(1) = 5.4, *p* = 0.02), with a decrease in the prevalence of these errors with age advancement in the TH group, while errors remained stable in the CI group. The opposite trend was observed for phonological level (χ^2^(1) = 10.3, *p* = 0.001). Errors involving verb forms were more frequent in the CI group with chronological age (β = −0.12, SD = 0.06, *t*(63) = −1.88, *p* = 0.05) and vocabulary level (β = −0.49, SD = 0.2, *t*(63) = −2.3, *p* = 0.02). An interaction effect between group and vocabulary level was noted, with errors decreasing as vocabulary increased in the CI group, while remaining stable in the TH group.

Children in the CI group make also more omissions of function words than children in the TH group. More specifically, CI children made significantly more determiner omissions across three covariates: chronological age (β = −3.2, SD = 0.96, *t*(63) = −3.4, *p* = 0.001), auditory age (β = −2.73, SD = 0.98, *t*(63) = −2.8, *p* = 0.007), and vocabulary level (β = −2.83, SD = 1, *t*(63) = −2.8, *p* = 0.007), and a significant negative correlation between error numbers and phonological level was observed. For preposition omissions, CI children made significantly more errors with auditory age (β = −0.05, SD = 0.02, *t*(63) = −2.2, *p* = 0.03) and phonological level (β = −0.07, SD = 0.02, *t*(63) = −2.4, *p* = 0.02) as covariates. Similarly, pronoun omissions were more frequent in the CI group with chronological age (β = −0.4, SD = 0.17, *t*(63) = −2.1, *p* = 0.04) as covariate, with a negative correlation observed between error numbers and phonological level. Finally, CI children made more errors involving diverse additions with chronological age (β = −0.07, SD = 0.03, *t*(63) = −1.9, *p* = 0.05), auditory age (β = −0.8, SD = 0.3, *t*(63) = −2.2, *p* = 0.03), vocabulary level (β = −0.8, SD = 0.4, *t*(63) = −2.2, *p* = 0.03), and phonological level (β = −0.1, SD = 0.04, *t*(63) = −2.5, *p* = 0.01).

### 3.3 Sentence/word-picture matching task

The scores obtained in the sentence/word-picture matching task were also compared between the CI and TH groups, with chronological and auditory ages, vocabulary levels, and phonological precision included as covariates. Table 5 summarizes the significance levels of the different subscores based on the covariates studied. Since no interaction effects were identified, they are not represented in Table 5.

**TABLE 5:** Summary of group effects (CI vs. TH) and target covariate effects (chronological age – CA, auditory age – AA, vocabulary level – VL, and phonological level – PL) for sentence/word-picture matching task subscores.

		Chronological age comparison		Auditory age comparison		Vocabulary level (VL) comparison		Phonological level (PL) comparison		Best comparison model
		Group effect	CA effect	Group effect	AA effect	Group effect	VL effect	Group effect	PL effect	
Grammatical opposition type	Number marking		*		*		*		***	PL
	Gender marking						*		**	PL
	Lexical minimal pairs	*							***	PL
Phonological opposition type	Nasal-oral	**	***		**				***	PL
	Oral-oral							*	**	PL
	Nasal-nasal								**	PL
	Addition						*		**	PL

Significant effects are indicated with *p < 0.05, **p < 0.005, or ***p < 0.001. The model with the lowest AIC (best model) is listed on the right.

A group effect favoring the TH group was observed for lexical minimal pairs (3.01 vs. 2.06; β = 0.95, SE = 0.46, *t*(63) = 2.1, *p* = 0.04) as well as for the nasal-oral opposition subscore (2.58 vs. 1.29; β = 1.3, SE = 0.4, *t*(63) = 3.2, *p* = 0.002) with chronological age as a covariate. However, this difference is no longer significant when controlling for the other covariates. Notably, phonological precision is strongly associated with each subscore, and models using this covariate exhibit the lowest AIC values.

### 3.4 Links between productive and receptive tasks with lexical and phonological scores

**FIGURE 3:**
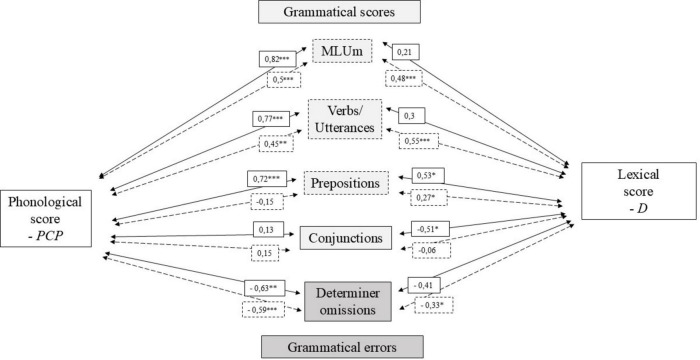
Pearson coefficients and associated significance levels between phonological scores (percentages of correct phonemes – PCP), lexical diversity scores (D index), and various grammatical measures. Solid arrows refer to the CI group, while dashed arrows refer to the TH group. Significant correlations are indicated with *p < 0.05, **p < 0.005, or ***p < 0.001).

The specific links between the different subscores and levels of phonological precision and vocabulary were finally compared between the two groups. Figure 3 represents the different correlation coefficients for the CI and TH groups between the phonological development score (percentages of correct phonemes) on one hand, and the averaged lexical diversity index of the two narratives (*D* scores) on the other hand, with the morphosyntactic development indices MLUm and the verb/utterances ratio, as well as with the number of nouns, prepositions and conjunctions as well as determiner omissions. This choice of verbal tense, function word, and error was made by selecting those that showed significant differences in terms of auditory status in the previous analyses of this study (see section “3.3 Sentence/word-picture matching task”).

The MLUm and V/U ratios both exhibited strong and significant correlations with the phonological score among children in the CI group and moderate correlations in the TH group. MLUm and V/U ratios showed moderate to strong correlations with the lexical diversity scores, but only within the TH group. For nouns and conjunctions, which were observed in greater numbers among the CI group, different trends were identified. The number of nouns was strongly negatively correlated with phonological scores in the CI group, while this same negative correlation was only observed with the lexical diversity scores in the TH group. Regarding conjunctions, children in the CI group exhibited moderate negative correlations between their conjunction scores and the lexical diversity scores. In contrast, prepositions, which were observed in significantly greater numbers in the narratives of TH group children, were strongly and positively correlated with phonological scores in the CI group and showed slight to moderate correlations with the lexical diversity scores in both groups. Additionally, phonological scores were strongly negatively correlated with the presence of determiner deletions in both groups and moderately negatively correlated with the lexical diversity scores in the TH group.

**FIGURE 4:**
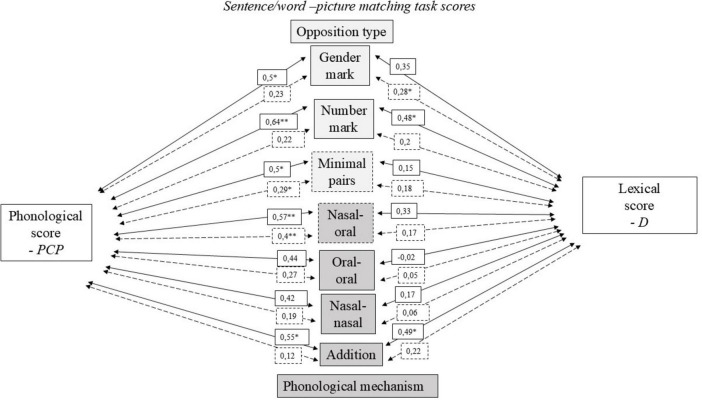
Pearson coefficients and associated significance levels between phonological scores (percentages of correct phonemes – PCP), lexical diversity scores (D index), and the various scores on the pointing task. Solid arrows refer to the CI group, while dashed arrows refer to the TH group. Significant correlations are indicated with *p < 0.05, **p < 0.005.

Figure 4 represented the different correlation scores for the CI and TH groups between the phonological development score, on one hand, and the averaged lexical diversity scores of the two narratives on the other hand, with the different sub-scores of the sentence/word-picture matching tasks.

Regarding the type of opposition, the phonological score was significantly and strongly correlated with the scores of number markers and the scores on minimal pairs and moderately correlated with the scores of gender markers among children in the CI group. Lexical diversity scores were moderately correlated with number marks score. Among children in the TH group, a moderate and significant link was observed between the percentages of correct phonemes and the score on minimal pairs, as well as between the lexical diversity scores and gender markers. Concerning scores according to the type of phonological mechanism, strong and significant links were observed between the phonological scores and the sub-scores related to the nasal-oral distinction and phonemic additions in the CI group. The sub-score related to phonemic additions was also moderately correlated with the phonological and lexical diversity scores. A moderate and significant link was observed between the phonological score and the sentence/word-picture matching task scores related to the distinction between nasal and oral vowels in the TH group.

**FIGURE 5:**
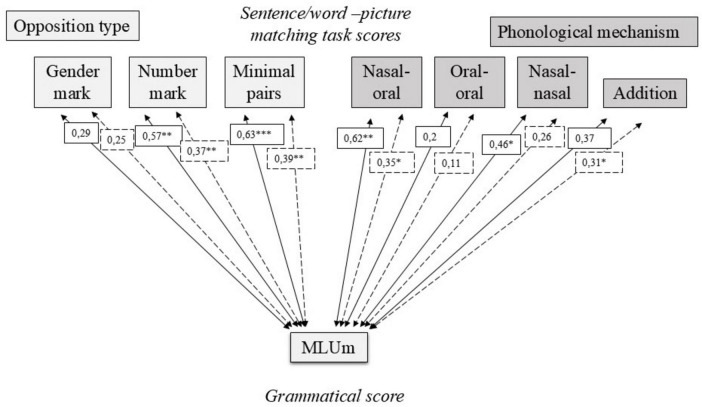
Pearson coefficients and associated significance levels between sentence/word-picture matching task scores and MLUm values. Solid arrows refer to the CI group, while dashed arrows refer to the TH group. Significant correlations are indicated with *p < 0.05, **p < 0.005, or ***p < 0.001.

Figure 5 presented the different subscores of the sentence/word-picture matching task with the morphosyntactic development index MLUm.

Moderate to strong significant positive associations were observed between number markers and minimal pairs with MLUm in both groups. Regarding phonological mechanisms, a strong and significant association was observed between the MLUm score and the subscore related to distinctions between nasal and oral vowels in children from the CI group, while this association was moderate in the TH group. A moderate association was also observed between MLUm and the subscore related to distinctions between nasal vowels in the CI group, as well as between MLUm and phonemic additions in the TH group. Note that similar relationships are generally observed between these different scores and the verb/utterance ratios (not presented in the figures), with the exception of the presence of additional strong (CI) and moderate (TH) correlations with gender marking (CI: 0.64; *p* = 0.003 – TH: 0.38; *p* = 0.008) and with the score implying distinction between two nasal vowels (CI: 0.38; *p* = 0.1 – TH: 0.36; *p* = 0.2). It is also interesting to note a moderate negative correlation between the omission of determiners and the sub-score related to distinctions between nasal and oral vowels in both groups (CI: −0.46; *p* = 0.04 – TH: −0.31; *p* = 0.03), while this same sub-score shows a significant negative correlation with the omission of determiners only in the TH group (CI: −0.33; *p* = 0.2 – TH: −0.37; *p* = 0.009).

## 4 Discussion

The present study aimed to investigate grammatical production skills through narrative production and morphophonological reception skills targeting grammatical markers and minimal pairs based on the distinction between French nasal and oral vowels in a group of children with CIs and their TH peers. Group comparisons were conducted while controlling for chronological and auditory ages, phonological skills (assessed through a picture-naming task), and lexical skills (measured using the lexical diversity index obtained from the narratives). The study also examined the specific relationships within the two groups between these skills and lexical/phonological levels, as well as the relationships between productive and receptive MS tasks.

### 4.1 Phonological and lexical skills

Lexical and phonological skills were first assessed within the two groups, taking into account both the chronological age of the children and their hearing age. Both types of comparisons led to the same conclusion: the performance of children with CI is lower than that of their TH peers of the same chronological and hearing age. Thus, children with CI exhibit phonological and lexical performance below what might be expected for children of the same age, even when considering their level of auditory experience.

Regarding the level of phonological development, this result is not surprising and supports the impact of perceptual limitations on the development of precise phonological representations. This finding aligns with the literature, which highlights increased difficulties in this component of language (Nittrouer et al., 2014, 2018; David et al., 2021; Romano et al., 2021). However, it was not expected to observe lower performance in terms of lexical diversity. Indeed, this finding might seem contradictory to the literature that suggests a preservation of lexical skills (Duchesne et al., 2009; Caselli et al., 2012; Rinaldi et al., 2013) justified by the perceptually and conceptually more salient nature of lexical elements in language (Joanisse and Seidenberg, 1998; Chiat, 2001). The results should also be contrasted with the study by Warner-Czyz et al. (2024), which showed that expressive vocabulary size, as assessed through parental reports in young children aged 17–30 months, exhibited a chronological delay relative to expected scores based on chronological age when compared to their peers with TH. However, this gap was compensated for when considering listening experience. It therefore appears that, unlike the early development of a lexicon, lexical diversity—when assessed through a narrative task—remains more challenging for children with CIs compared to their TH peers, even when auditory experience is equivalent. However, it should be noted that a similar result was obtained in narrative productions by Le Normand and Thai-Van (2023). Methodological differences could explain this discrepancy between studies, as those reporting a selective grammatical deficit typically used formal language assessment tests, which mainly involved tasks such as naming or picture identification to assess receptive and/or productive lexical levels. In contrast, the language mechanisms involved in narrative production are more complex and closer to an ecological language production situation. Our present data corroborate Le Normand and Thai-Van’s findings, suggesting that lexical development might also pose long-term challenges for children with CI.

It is worth highlighting that both groups showed improved performance with increasing chronological and hearing age, indicating a maturation effect on these performances regardless of auditory status. Furthermore, the gap between the two groups is more pronounced for phonological scores, possibly indicating a greater vulnerability for this language component among children in the CI group.

### 4.2 Narrative productions skills

The analysis of narrative productions revealed significantly lower scores in developmental cues in CI children, specifically lower MLU a lower V/U ratio, when controlling for chronological ages. Lower MLU values in CI groups compared to chronological age-matched peers have already been reported in the literature (Szagun, 2001; Hansson et al., 2017; Nittrouer et al., 2018; Majorano et al., 2024), which has led to conclusions about increased vulnerability in MS skills among CI children in production. The V/U ratio highlights the number of complete sentences, meaning those that include a main verb. The ratio for the CI group is significantly lower than that of the TH group, indicating a higher proportion of verbless utterances. These results have also been observed in a study of Italian-speaking children (Majorano et al., 2024), and further supports the observation of grammatical difficulties in these children. This advantage of the TH group children, however, disappears once the children are compared while accounting for their hearing ages and linguistic levels, as no statistically significant differences were observed when using hearing age and vocabulary and phonological levels as covariates. It therefore seems that, with an equivalent experience of auditory stimulation (at least through the activation of the first CI), the children produce utterances containing as many grammatical morphemes (MLU) and generate as many utterances with a conjugated verb (V/U ratios). Similar findings were made regarding hearing age (Caselli et al., 2012) and vocabulary level (Jung and Ertmer, 2018) in relation to MLU values. With respect to the matching in terms of phonological precision, to our knowledge, these are the first data on this subject, supporting the idea that better phonological performance is associated with increased MS performance and performance as strong as that of TH peers with similar phonological levels. These findings suggest a global positive linguistic effect linked to exposure to oral linguistic input, where greater auditory experience is associated with more opportunities to enhance phonological and lexical skills, thus leading to better MS performance. This assumption aligns with the literature proposing a commensurate development of lexical and phonological components (Stoel-Gammon, 2011; Sosa and Stoel-Gammon, 2012). It should be noted, however, that the models involving the level of phonological precision were the most predictive of the MLU and V/U ratios, indicating a predominant role of this language component in MS performance, as will be discussed below.

However, despite equivalent MLU values during pairings based on hearing ages and linguistic levels, the analysis of function words revealed differences between our groups in the distribution of certain function words. Specifically, a higher percentage of prepositions, reflexive pronouns, and possessive determiners was observed in the productions of the TH group, even when accounting for chronological age, hearing age, and vocabulary and phonological levels. A higher prevalence of object pronouns and article determiners was also noted when controlling vocabulary level. In contrast, subject pronouns, as well as adjectives and adverbs, were produced similarly in both groups. However, conjunctions were observed in greater proportions in the CI group when controlling for all four covariates. This differentiated effect of auditory status on the acquisition of function words echoes findings observed in French in younger children (Le Normand, 2004; Le Normand and Thai-Van, 2023), which had been attributed to the more or less lexicalizable and/or accented status of certain function words, giving them a perceptual advantage. In the same vein, within the early lexicon of young children, Warner-Czyz et al. (2024) found a significantly higher proportion of nouns compared to verbs among children with hearing loss using hearing aids or CIs. The authors suggested a facilitative effect in noun acquisition, which could be attributed to the greater ease with which nouns can be explicitly labeled. The study of verb tenses and moods produced by the children revealed a higher usage of the conditional, past simple, and future tenses in the TH group when controlling for chronological age. However, this advantage was no longer significant when controlling for hearing age and phonological and lexical levels. In contrast, the children in the CI group showed a higher prevalence in the use of the past perfect tense, regardless of the covariate. Children in the CI group, therefore, exhibited less variety in the use of tenses and moods compared to peers of the same chronological age but demonstrated equivalence to peers with the same hearing age and phonological/lexical levels. Auditory experience and linguistic levels again appear to be associated with better mastery of verbal morphology. However, it is worth noting the higher use of the past perfect: the use of an auxiliary may be more perceptually salient in the past perfect tense, with a past participle that is phonologically stable. The fact that, despite controlling for age effects (chronological and hearing) and linguistic levels (both phonological and lexical), these differences in the distribution of function words and verb tenses/moods persist suggests that they may be linked to a specific atypical development in children with CIs. This could be attributed to the perceptual limitations of the CI.

This observation is supported by the fact that the analysis of errors revealed more errors in nominal agreement (within the determiner + noun relationship) in CI children, as well as more deletions of determiners and prepositions, along with a greater variety of additions and errors in verbal tense forms, even when controlling for all covariates. Morphological errors related to gender marking have also been previously observed in children with CI in French (Le Normand and Thai-Van, 2023) as well as in other languages (Szagun, 2004; Moreno-Torres and Torres, 2008), with difficulties in grammatical morphology manifesting as morpheme substitutions. These errors in verbal or nominal agreement, manifested through substitutions, suggest a potential grammatical deficit and/or specific processing difficulties. In our sample, the children also made a number of omissions of function words, particularly prepositions and determiners. Determiner omissions have been suggested as a sign of perceptual difficulties (Moreno-Torres and Torres, 2008) and/or prosodic difficulties (Le Normand and Moreno-Torres, 2014; Le Normand and Thai-Van, 2023).

An integrative explanatory approach for these various findings is provided by Moreno-Torres and Moruno-López (2014). In that study, the authors observed different error profiles in suprasegmental aspects compared to hearing peers, which may suggest a tendency of CI children to reproduce correct syllabic sequences of the language without accessing the complete prosodic structure (perception of prosodic variations related to F0). The authors integrate their various findings into theoretical models of speech motor control (Hickok, 2012), proposing that the segmental elements of speech rely on both auditory-motor and somatosensory-motor cues. While the former cues are the first to be employed in development due to early guidance by auditory feedback, the latter cues are used later during the initial stages of speech production (babbling, first words). The literature suggests to Moreno-Torres and Moruno-López (2014) that children with CIs are more efficient in using auditory-motor cues, allowing them to quickly acquire a certain number of segments and the syllabic structure of their language after implantation. However, they face difficulties arising from their perceptual limitations, explaining the challenges in mastering certain phonological features. In contrast, they find it harder to rely on somatosensory-motor cues, which are more closely associated with phonemic units and implicit learning, possibly explaining the increased difficulty in perceiving consonantal and prosodic units. In this context, the authors propose a deficit in the use of the dorsal stream of the brain, based on the dual-stream processing model (Hickok and Poeppel, 2004; Friederici, 2012). According to this theory, the dorsal stream is associated with auditory-motor motor integration responsible for segmental-level processing, while the ventral stream, associated with auditory-motor conceptual integration, is used for semantic-lexical access. This hypothesis also highlights difficulties in implicit learning associated with the dorsal stream deficit, which may explain the increased dependence on explicit teaching and the significant inter-individual variability characteristic of this population. This explanatory framework is fully consistent with the observations of the present study: children with CIs struggle more to acquire function words, whose conceptual representation is less prominent and less easily accessible through explicit teaching. This leads to difficulties such as substitutions and omissions of morphemes, also due to incomplete perception of the prosodic elements of the language.

### 4.3 Processing grammatical and lexical morphemes in nasal-oral vowel morphophonological opposition

In the morpheme identification task, an effect of auditory status was observed only for the lexical minimal pairs and the score that included items where the morphemic opposition was carried by nasal vs. oral vowels when controlling for chronological age. This finding supports the hypothesis of increased difficulty in processing certain phonological features, specifically vowel nasality, which significantly impacts morphemic processing. These results align with previously observed difficulties in the identification and discrimination of nasal and oral vowels in children with CIs (Fagniart et al., 2024a). Given that the minimal pairs primarily involved oppositions between nasal and oral vowels, it is unsurprising that the score associated with minimal pairs was generally lower for children with CIs compared to TH children. However, as observed with MLU and V/U ratios, the performances of the CI group were no longer statistically inferior when controlling for hearing age and phonological and lexical levels. With equivalent auditory experience and linguistic levels, it seems that the specific perceptual difficulties associated with vowel nasality have less impact on morphemic processing skills.

One way to explain these results would be that the perceptual difficulties related to vowel nasality have diminished, allowing for better discrimination of nasal-oral vowel segments. It is worth recalling that the nasal timbre of nasal vowels, as opposed to the oral timbre of oral vowels in French, is particularly reliant on acoustic information that requires optimal spectral resolution skills. While these spectral resolution skills develop progressively from childhood to adolescence in TH children (Jahn et al., 2022), numerous studies report significant difficulties in children with CIs, with little improvement as they age, reflecting characteristic perceptual limitations (Horn et al., 2017; Gifford et al., 2018; Landsberger et al., 2018). This is consistent with lower performance in the identification and discrimination of nasal-oral vowels in children aged 5–12 years (Fagniart et al., 2025), with no significant effect of chronological or hearing age on performance. However, a recent study highlighted a moderate link between spectral modulation detection thresholds and chronological age in a group of children aged 5–13 years, despite significantly lower performance compared to their TH peers (DeFreese et al., 2024). The effects of maturation on these fine spectral processing skills remain inconclusive in the literature and warrant further investigation to determine their developmental trajectory in children with CIs. The data from this study could support the notion that these skills may evolve with auditory and linguistic experience. It is important to emphasize that the linguistic material used in this study consisted of real words and syntagms, which offers various perceptual advantages compared to studies on spectral resolution or discrimination/identification of nasal-oral vowels using synthetic sounds, isolated vowels, or pseudowords. While these latter approaches are controlled to require isolated processing of the target vowel/sound, real words benefit from stored phonological representations in memory and include an associated phonetic context. This context provides coarticulatory effects and supplementary acoustic information that are potentially more accessible through CI coding, such as formant transitions with adjacent consonants and differences in amplitude or segmental duration. It is thus possible that, with auditory and linguistic experience, children in the CI group developed greater compensatory strategies for their initial perceptual difficulties, enabling them to rely more effectively on perceptual cues better encoded by the CI to refine their phonological representations of challenging sounds. This observation has been reported in studies investigating the perception and production of nasal-oral vowels, where the most proficient children appeared to rely more on temporal cues in perception (Fagniart et al., 2024a) and visual mechanisms in production (Fagniart et al., 2025).

### 4.4 Is there a link between lexical, phonological, and grammatical abilities?

The relationships between the phonological, morphosyntactic, and lexical components of language among our groups of children showed different profiles consistent with specific difficulties encountered by CI children. Indeed, the two groups appear to exhibit differentiated relationships between the various components and MS scores: while children in the TH group tend to have their scores evolve more jointly (associations between phonological, lexical, and MS production performance), reflecting concurrent global improvements in linguistic performance, children in the CI group with higher developmental MS scores and greater prevalence of MS complexity markers (use of prepositions, appropriate presence of determiners) are more strongly, or even exclusively, associated with phonological scores compared to lexical scores. These specific links among CI children between their phonological and morphosyntactic abilities are consistent with phonological theories of MS difficulties proposed to explain the language disorders encountered by children with SLI (Leonard et al., 1992; Joanisse and Seidenberg, 1998; Chiat, 2001; Parisse and Maillart, 2008). The phonological difficulties presented here by children with CI, stemming from the perceptual limitations of the CI, more severely affect MS development due to the lack of perceptual and conceptual prominence of grammatical elements in the linguistic input.

Regarding the task of processing grammatical and lexical morphemes, a specific link between the subscores and phonological skills was also observed in the CI group, indicating a specific relationship between phonological and morphophonological processing levels, not mediated by differences in overall language development. These observations corroborate the links previously observed between syntactic comprehension and phonological scores among Swedish children (Hansson et al., 2017). However, links were found between the lexical diversity index and gender markings in TH children, as well as number markings in CI children, which may reflect effects related to differences in general linguistic level for these scores. The correlation between the phonemic addition score and both phonological and lexical scores in the CI group further supports this.

The study of the relationships between the various subscores of the morphemic processing task and MS production scores also highlighted stronger links between the scores associated with the processing of nasal vowels (nasal-oral and nasal-nasal) and the MLUm and V/U ratios in the CI group. Children who are proficient at discriminating grammatical and lexical items distinguished by nasal vowels, either from each other or from oral vowels, also tend to have higher MLUm values and more complete sentences (containing a verb). Additionally, a link is observed between the processing of nasal and oral vowels and a reduction in the number of determiner in both groups. This is not surprising either: both skills can be attributed to better perceptual processing at both the segmental level (discrimination of nasal and oral vowels—fine spectral processing) and the suprasegmental level (adequate presence of determiners) in line with Moreno-Torres and Moruno-López (2014). These various results seem to support the hypothesis of variability in MS performance that can be explained by different degrees of perceptual processing in children with CIs.

### 4.5 Limitations

Although this study has allowed for the formulation of numerous findings in connection with the literature, it suffers from various limitations that are important to put into perspective. The first potential bias of the study is the limited sample size, which is characteristic of studies involving CI users. It is indeed complex to recruit a relatively homogeneous sample and to control all the key variables (rehabilitation methods, implantation age, etc.). The inter-individual variability characteristic of the CI population complicates the interpretation of the various observed effects, especially given the small sample sizes. Moreover, it would be interesting to replicate this type of study to investigate different language components jointly but in a longitudinal manner, to obtain more reliable developmental data. Different environmental variables could be controlled in studies of this type, such as parental involvement or parenting practices known to have a positive impact on language development.

## 5 Conclusion

This study examined the connections between phonological and morphosyntactic components in children with CI, focusing on their processing skills related to the phonological feature of vowel nasality in French.

Several findings emerged:

– Children in the CI group exhibited significantly lower performance compared to their peers with TH of equivalent chronological age. This was evident in their MS production (e.g., shorter MLUm, more non-verbal utterances, less complex function words, and verbal morphology) and in their processing of grammatical and lexical morphemes carried by nasal and oral vowels.
– When accounting for auditory age or phonological/lexical levels, differences in MS indices (e.g., MLUm and V/U) and morphosyntactic processing scores were no longer statistically significant between groups. With similar auditory experience or phonological/lexical levels, the effect of auditory status disappears, suggesting a capacity to compensate for the initial perceptual limitations associated with CI.
– A distinct pattern in the use of function words was observed, with fewer complex function words and verb tenses, alongside more frequent errors (e.g., addition/omission of function words and misuse errors). These findings reflect specific difficulties likely attributable to the perceptual limitations of CI.
– A specific link was found between MS skills (both in production and morphemic processing) and phonological accuracy within the CI group. This stronger interdependence between levels could partially explain the significant inter-individual variability observed in MS development among children with CI, as their MS development is highly dependent on phonological skills, which are particularly at risk in this population.

These findings underscore the importance of early efforts to establish a stable and complete phonological system by employing targeted rehabilitation methods tailored to the perceptual limitations of children with CI. Indeed, under-specified phonological representations, coupled with a processing approach more focused on salient lexical elements, can potentially lead to substantial long-term linguistic challenges.

## Data Availability

The raw data supporting the conclusions of this article will be made available by the authors, without undue reservation.
