# Supplementary material for: Morphosyntactic production and processing skills in relation to age effects and lexical-phonological levels among children with cochlear implants and typically hearing peers: a focus on vowel nasality
Source: Front Hum Neurosci. 2025 Feb 26;19:1528388. doi: 10.3389/fnhum.2025.1528388 (PMC11897031; doi:10.3389/fnhum.2025.1528388)
Supplement: Supplementary file 1 [file Table_1.DOCX]

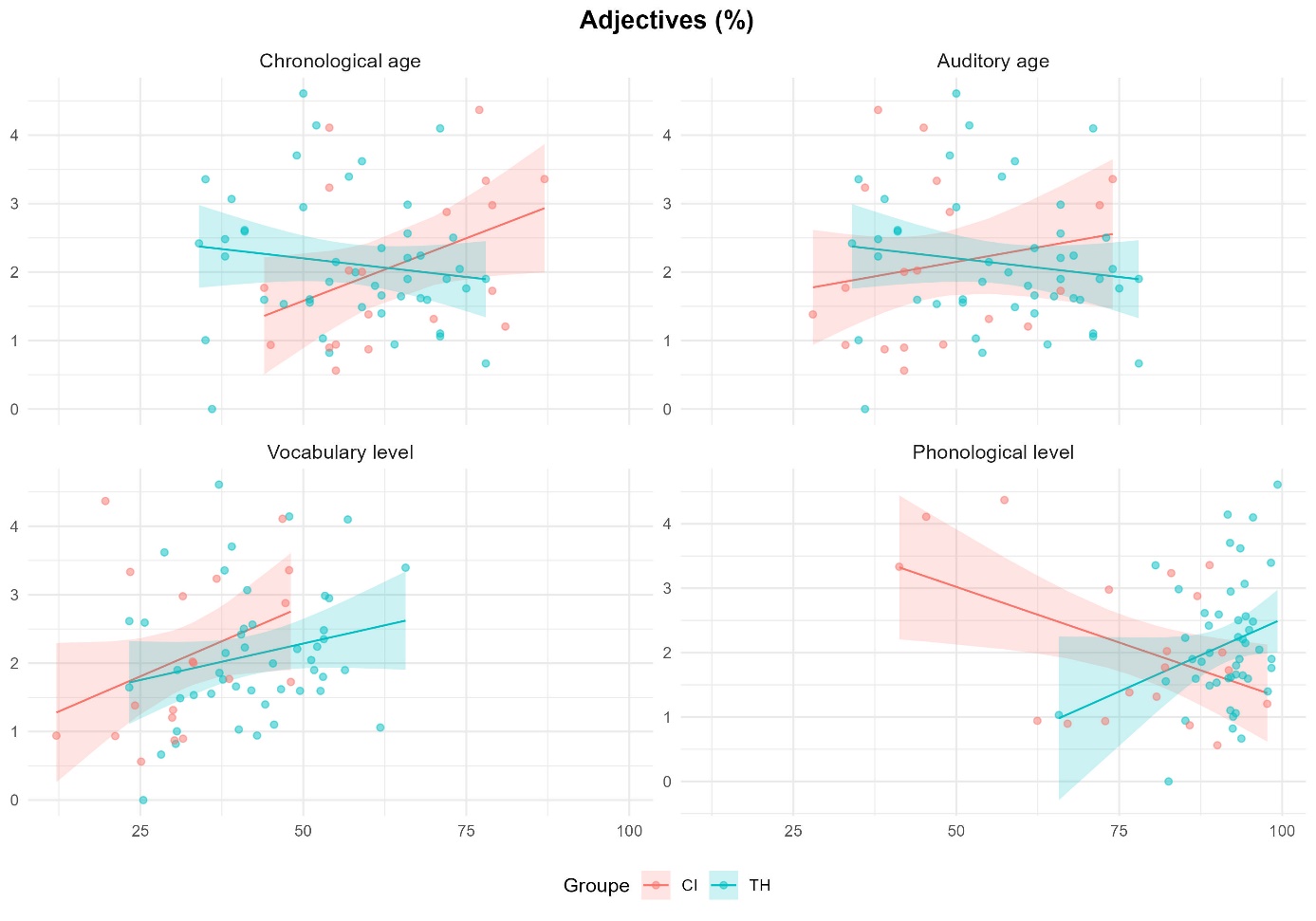


**Supplementary 1.1: Scatterplots of percentage scores percentages of adjectives as a function of chronological age (top left), auditory age (top right) in months, vocabulary (bottom left), and phonological level (bottom right) for CI (red) and TH (blue) groups. Regression lines with 95% prediction intervals, based on the tested mixed models, are included.**


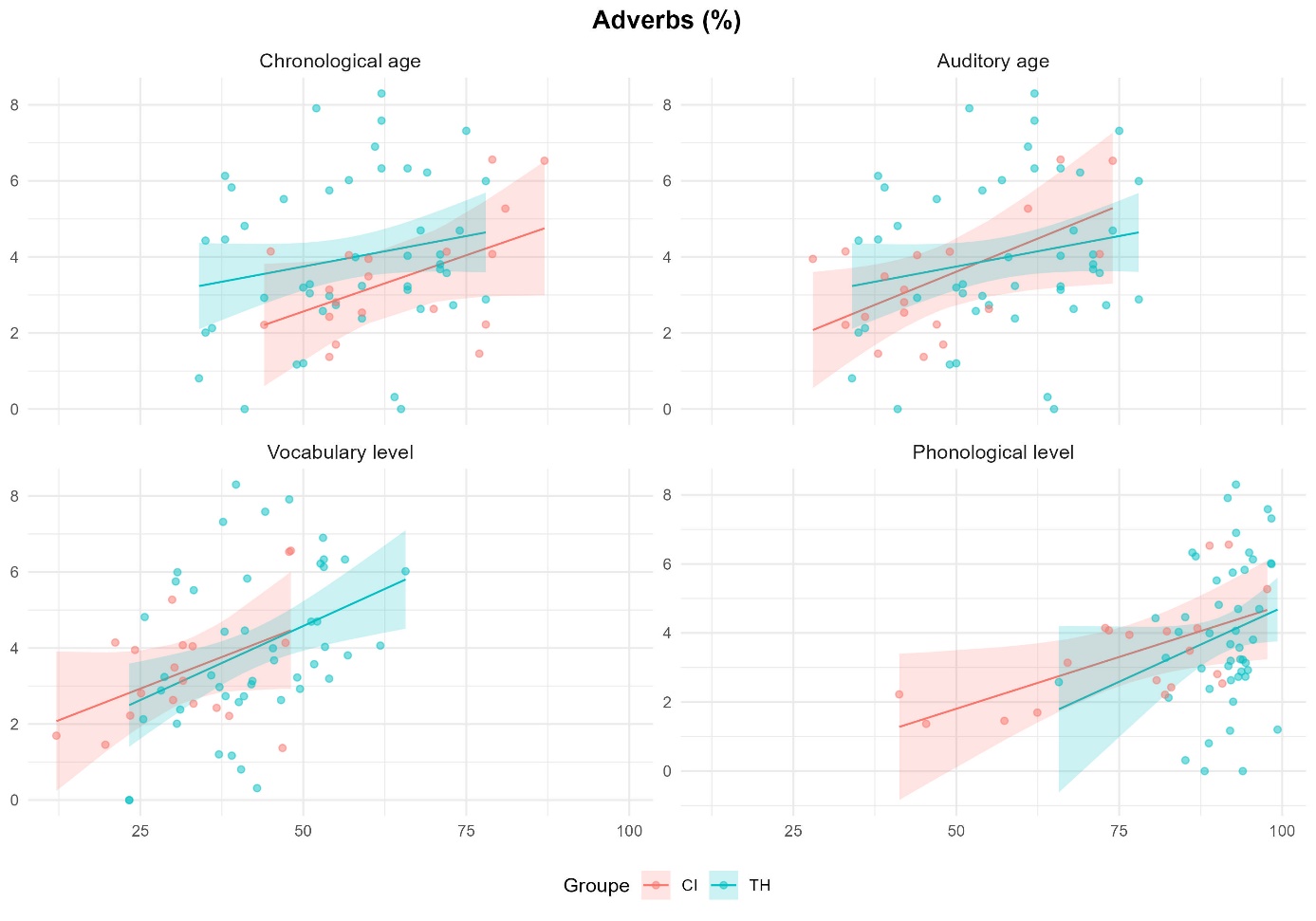


**Supplementary 1.2: Scatterplots of percentage scores percentages of adverbs as a function of chronological age (top left), auditory age (top right) in months, vocabulary (bottom left), and phonological level (bottom right) for CI (red) and TH (blue) groups. Regression lines with 95% prediction intervals, based on the tested mixed models, are included.**


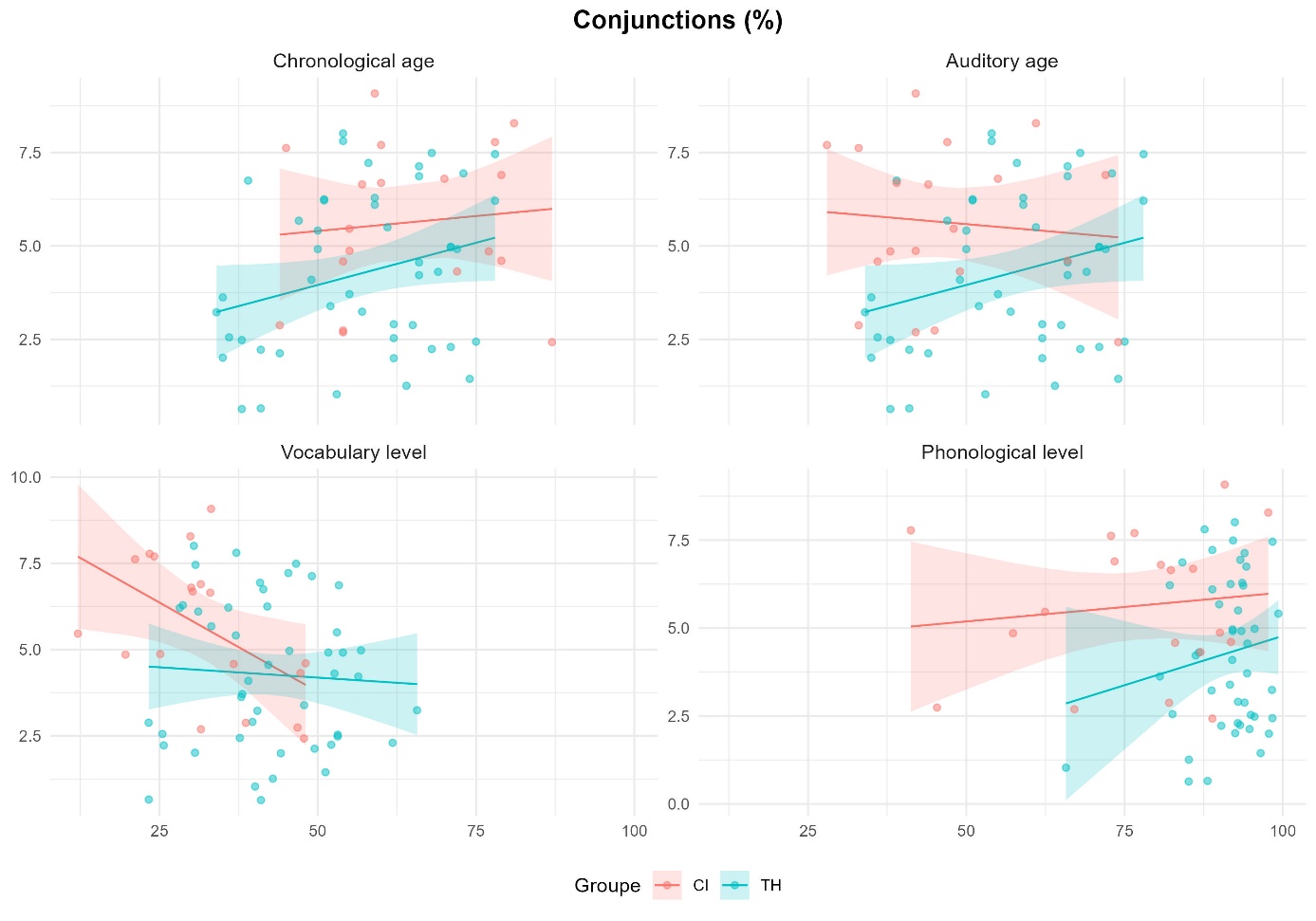


**Supplementary 1.3: Scatterplots of percentage scores percentages of conjunctions as a function of chronological age (top left), auditory age (top right) in months, vocabulary (bottom left), and phonological level (bottom right) for CI (red) and TH (blue) groups. Regression lines with 95% prediction intervals, based on the tested mixed models, are included.**


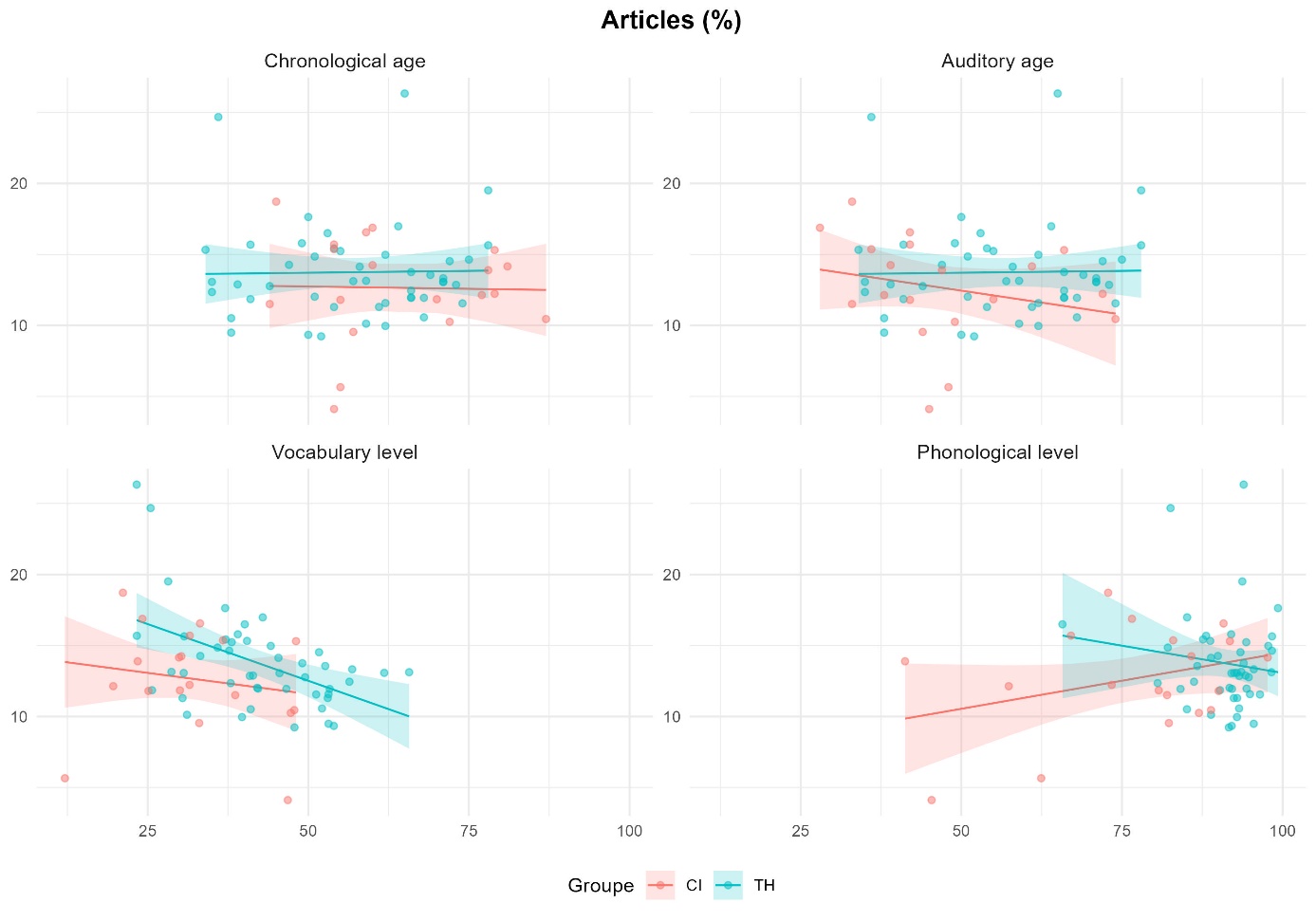


**Supplementary 1.4: Scatterplots of percentage scores percentages of article determiners as a function of chronological age (top left), auditory age (top right) in months, vocabulary (bottom left), and phonological level (bottom right) for CI (red) and TH (blue) groups. Regression lines with 95% prediction intervals, based on the tested mixed models, are included.**


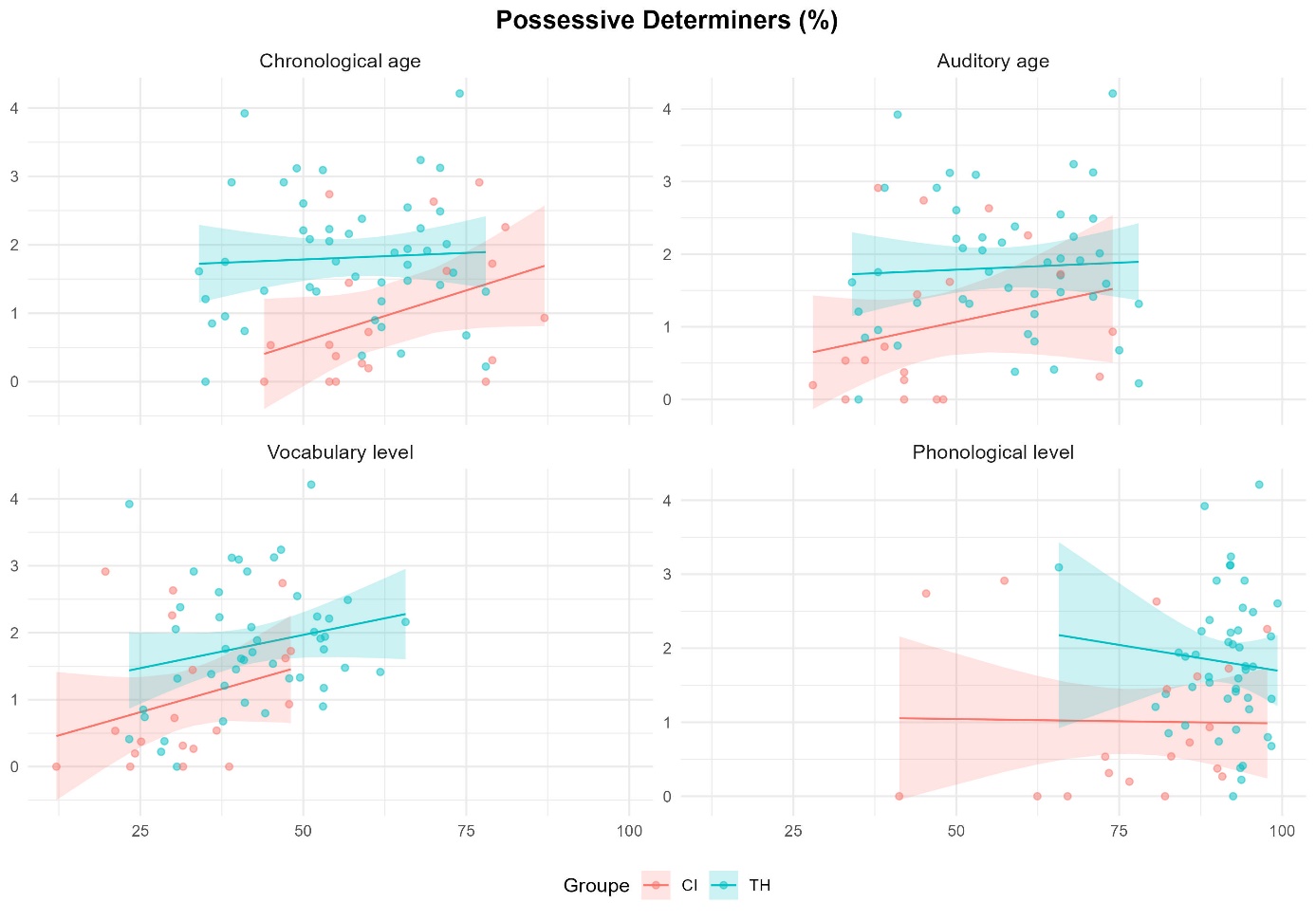


**Supplementary 1.5: Scatterplots of percentage scores percentages of possessive determiners as a function of chronological age (top left), auditory age (top right) in months, vocabulary (bottom left), and phonological level (bottom right) for CI (red) and TH (blue) groups. Regression lines with 95% prediction intervals, based on the tested mixed models, are included.**

4
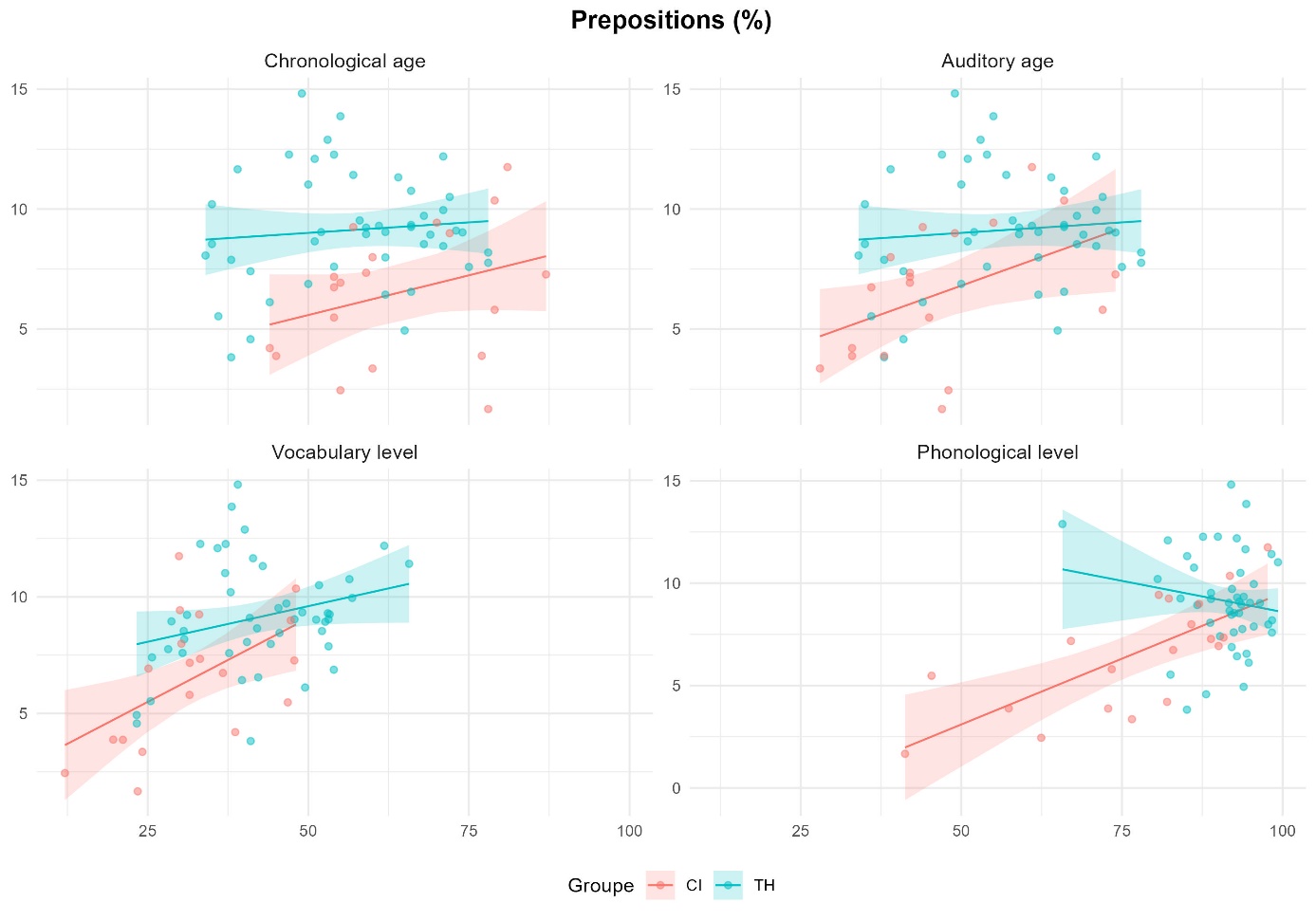


**Supplementary 1.6: Scatterplots of percentage scores percentages of prepositions as a function of chronological age (top left), auditory age (top right) in months, vocabulary (bottom left), and phonological level (bottom right) for CI (red) and TH (blue) groups. Regression lines with 95% prediction intervals, based on the tested mixed models, are included.**


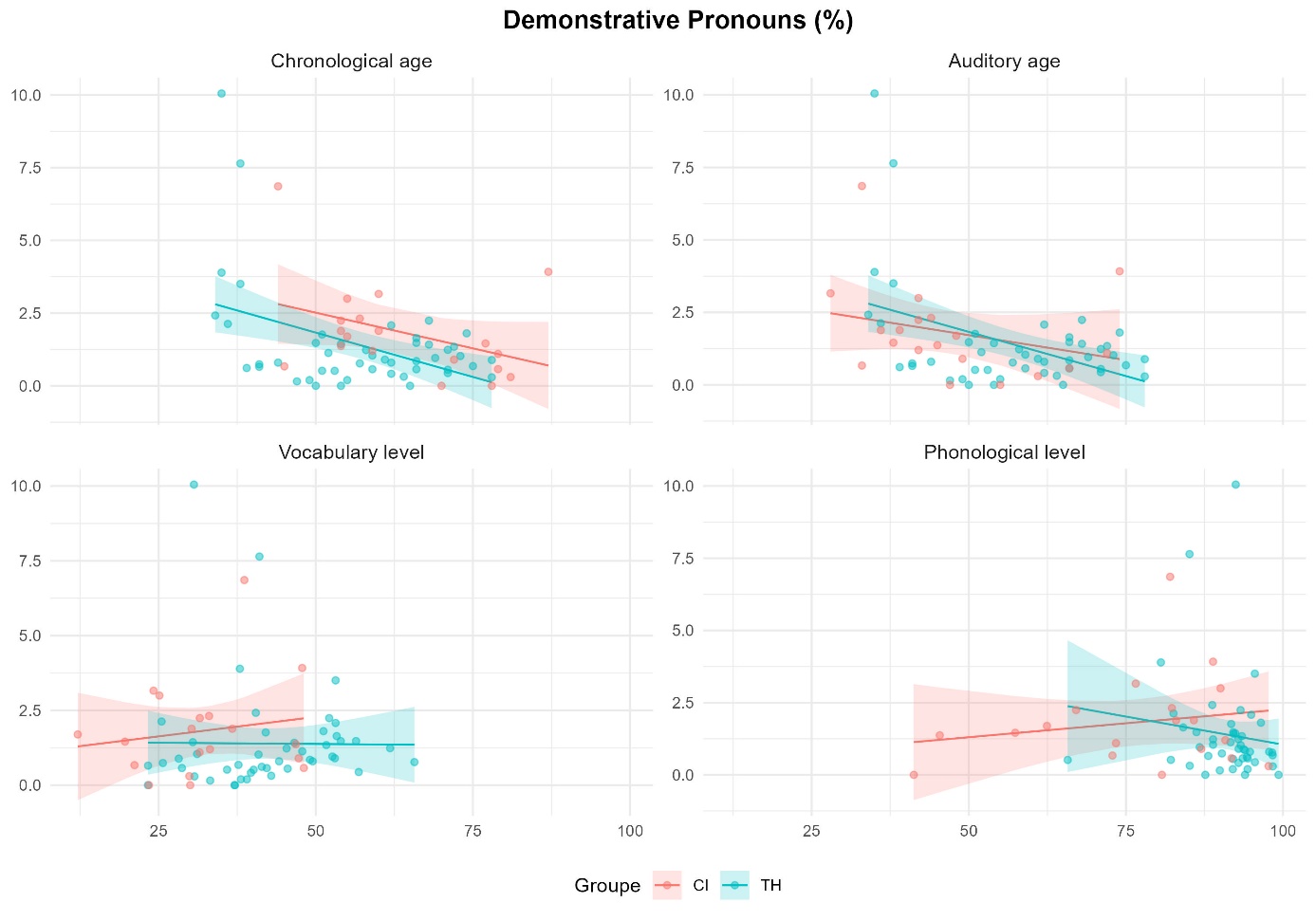
 **Supplementary 1.7: Scatterplots of percentage scores percentages of demonstrative pronouns as a function of chronological age (top left), auditory age (top right) in months, vocabulary (bottom left), and phonological level (bottom right) for CI (red) and TH (blue) groups. Regression lines with 95% prediction intervals, based on the tested mixed models, are included.**


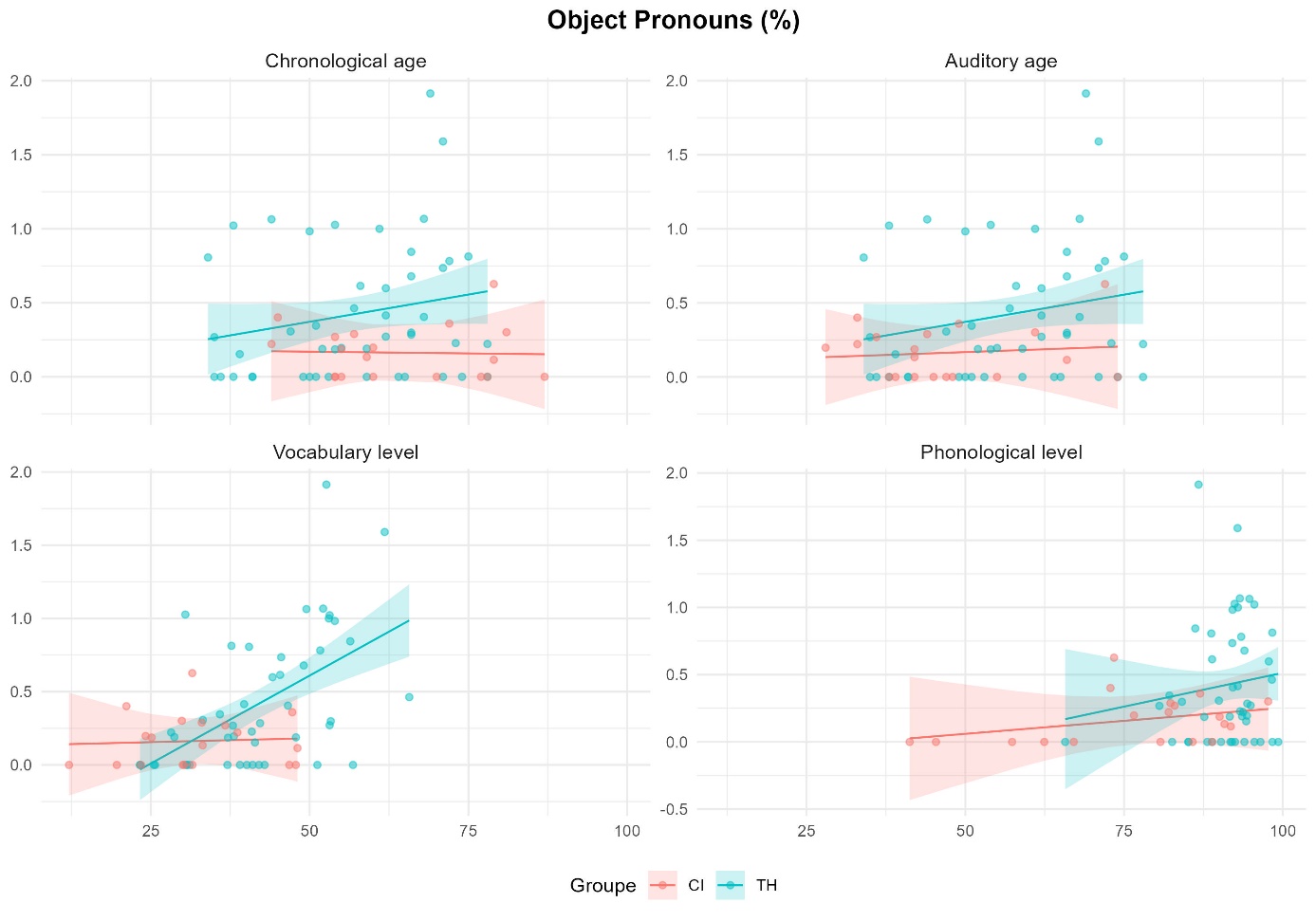


**Supplementary 1.8: Scatterplots of percentage scores percentages of object pronouns as a function of chronological age (top left), auditory age (top right) in months, vocabulary (bottom left), and phonological level (bottom right) for CI (red) and TH (blue) groups. Regression lines with 95% prediction intervals, based on the tested mixed models, are included.**


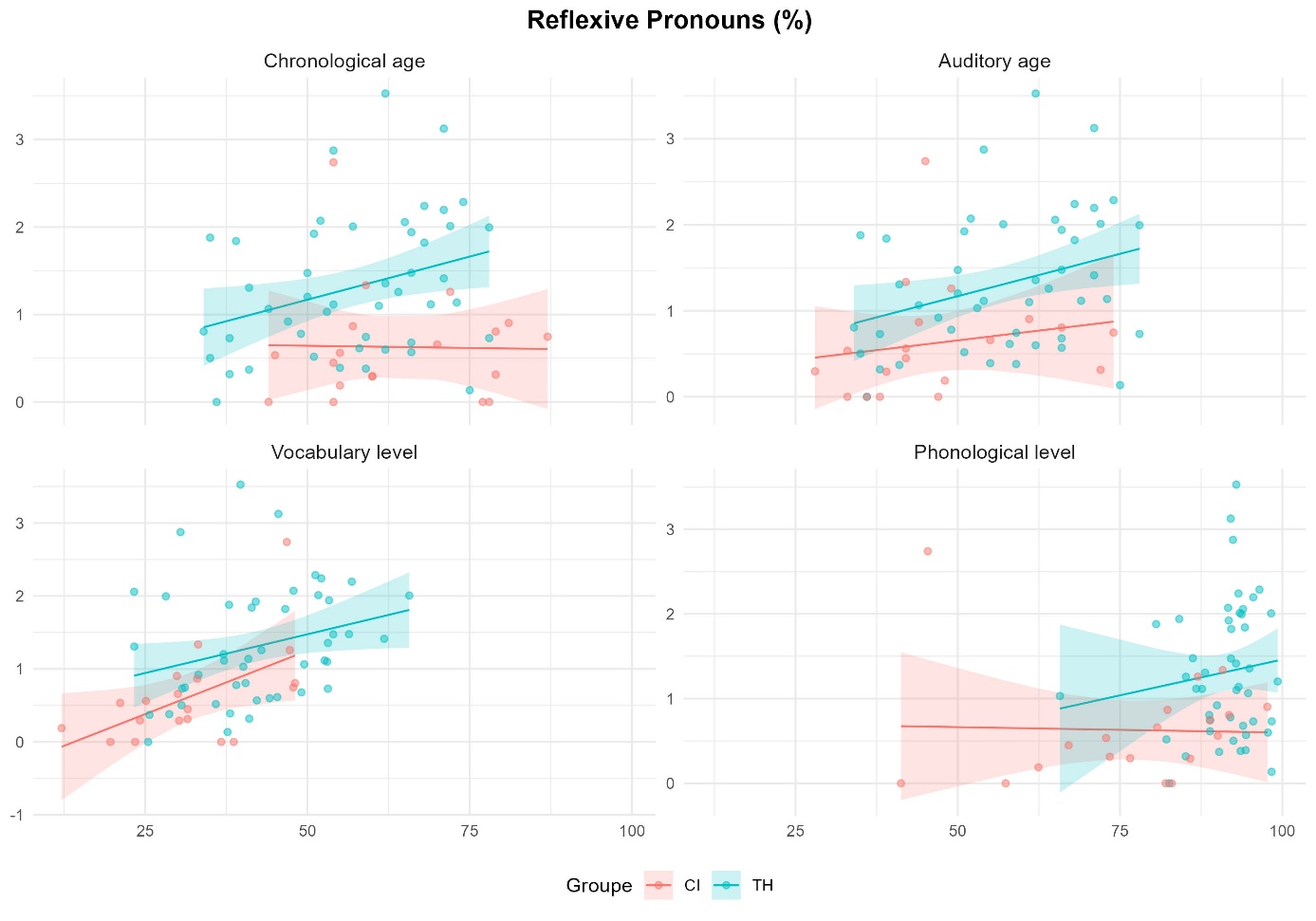


**Supplementary 1.9: Scatterplots of percentage scores percentages of reflexive pronouns as a function of chronological age (top left), auditory age (top right) in months, vocabulary (bottom left), and phonological level (bottom right) for CI (red) and TH (blue) groups. Regression lines with 95% prediction intervals, based on the tested mixed models, are included.**


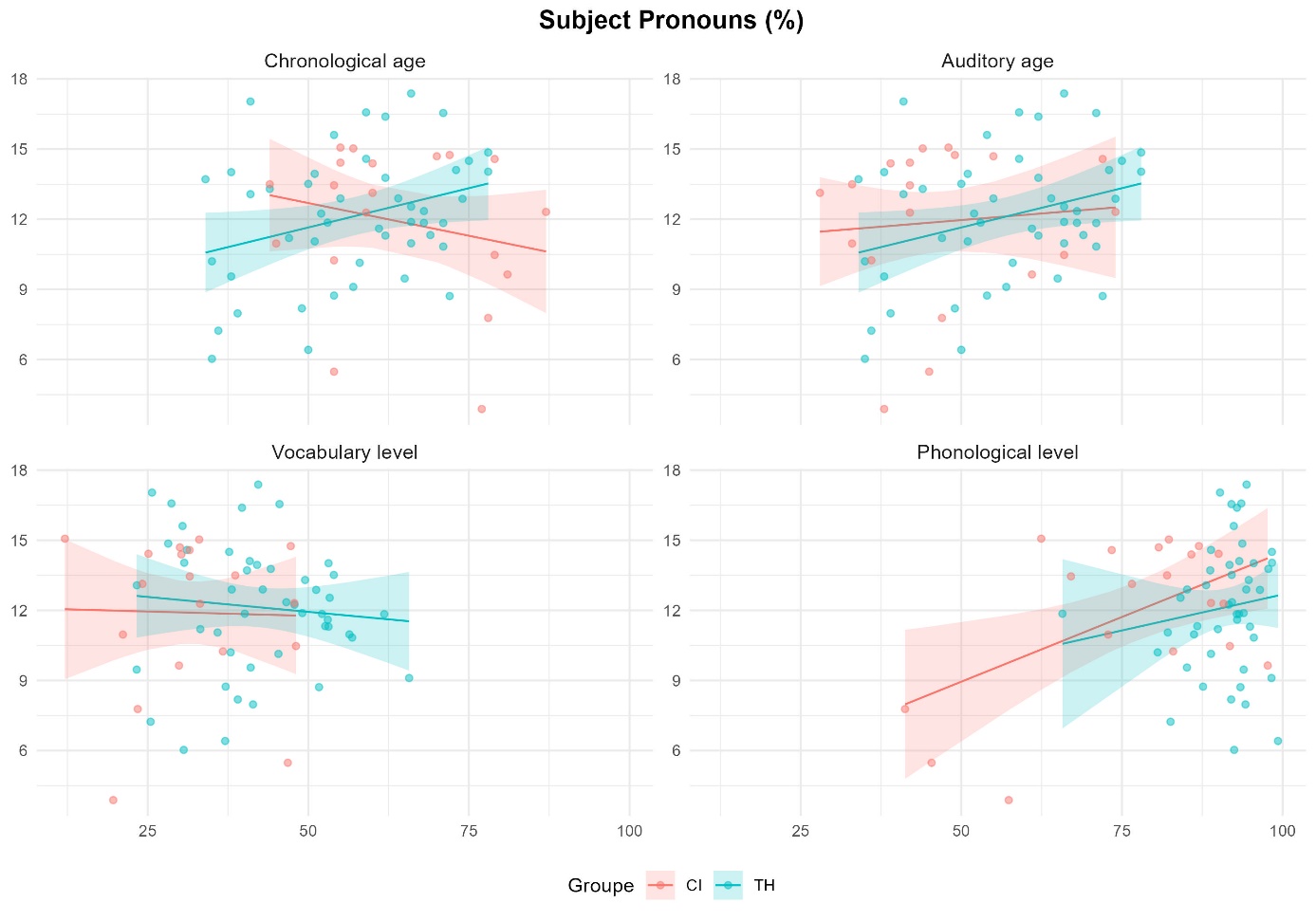


**Supplementary 1.10: Scatterplots of percentage scores percentages of subject pronouns as a function of chronological age (top left), auditory age (top right) in months, vocabulary (bottom left), and phonological level (bottom right) for CI (red) and TH (blue) groups. Regression lines with 95% prediction intervals, based on the tested mixed models, are included.**
