# Supplementary material for: Morphosyntactic production and processing skills in relation to age effects and lexical-phonological levels among children with cochlear implants and typically hearing peers: a focus on vowel nasality
Source: Front Hum Neurosci. 2025 Feb 26;19:1528388. doi: 10.3389/fnhum.2025.1528388 (PMC11897031; doi:10.3389/fnhum.2025.1528388)
Supplement: Supplementary file 2 [file Table_2.DOCX]

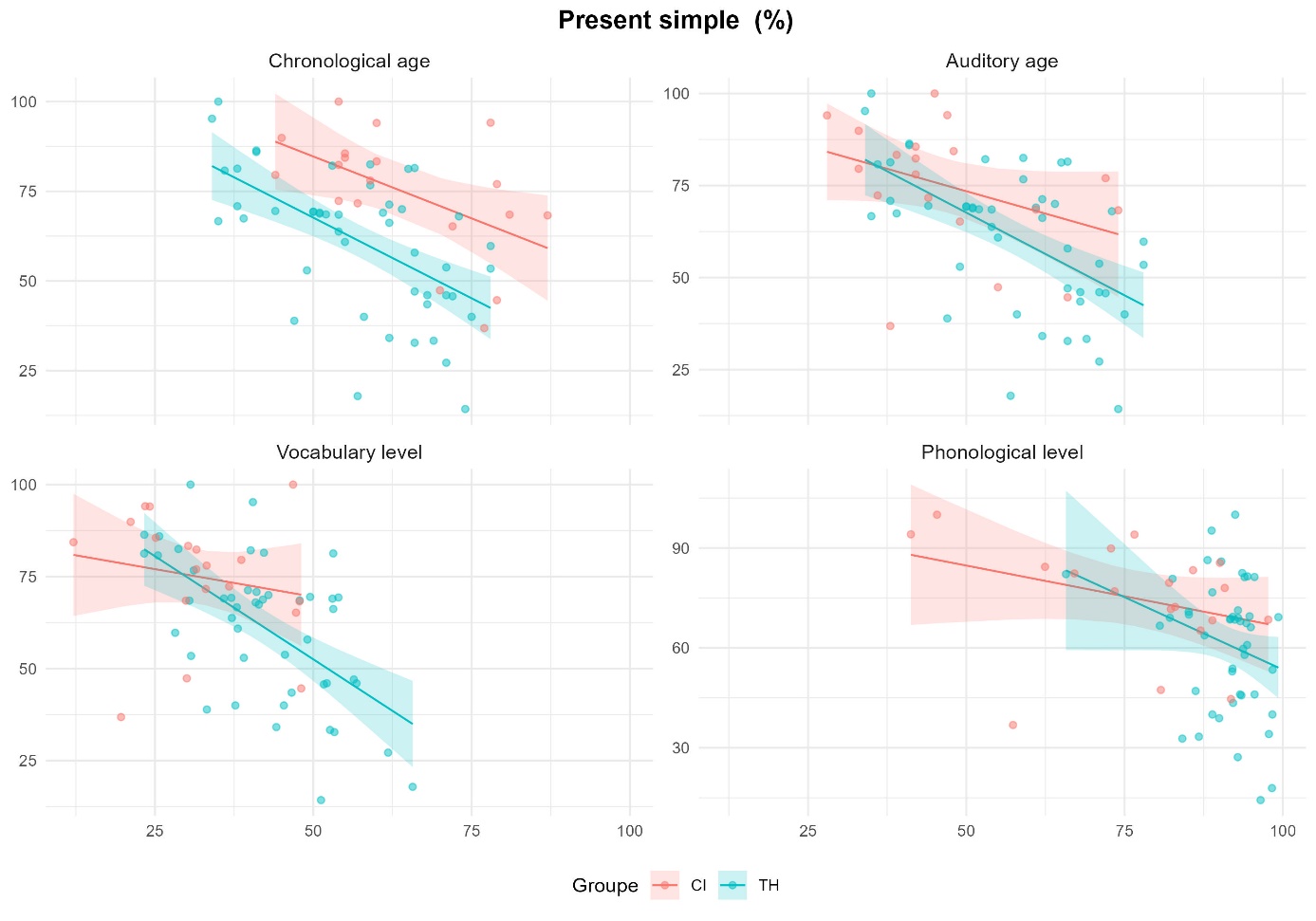


**Supplementary 2.1: Scatterplots of percentage scores percentages of present simples as a function of chronological age (top left), auditory age (top right) in months, vocabulary (bottom left), and phonological level (bottom right) for CI (red) and TH (blue) groups. Regression lines with 95% prediction intervals, based on the tested mixed models, are included.**


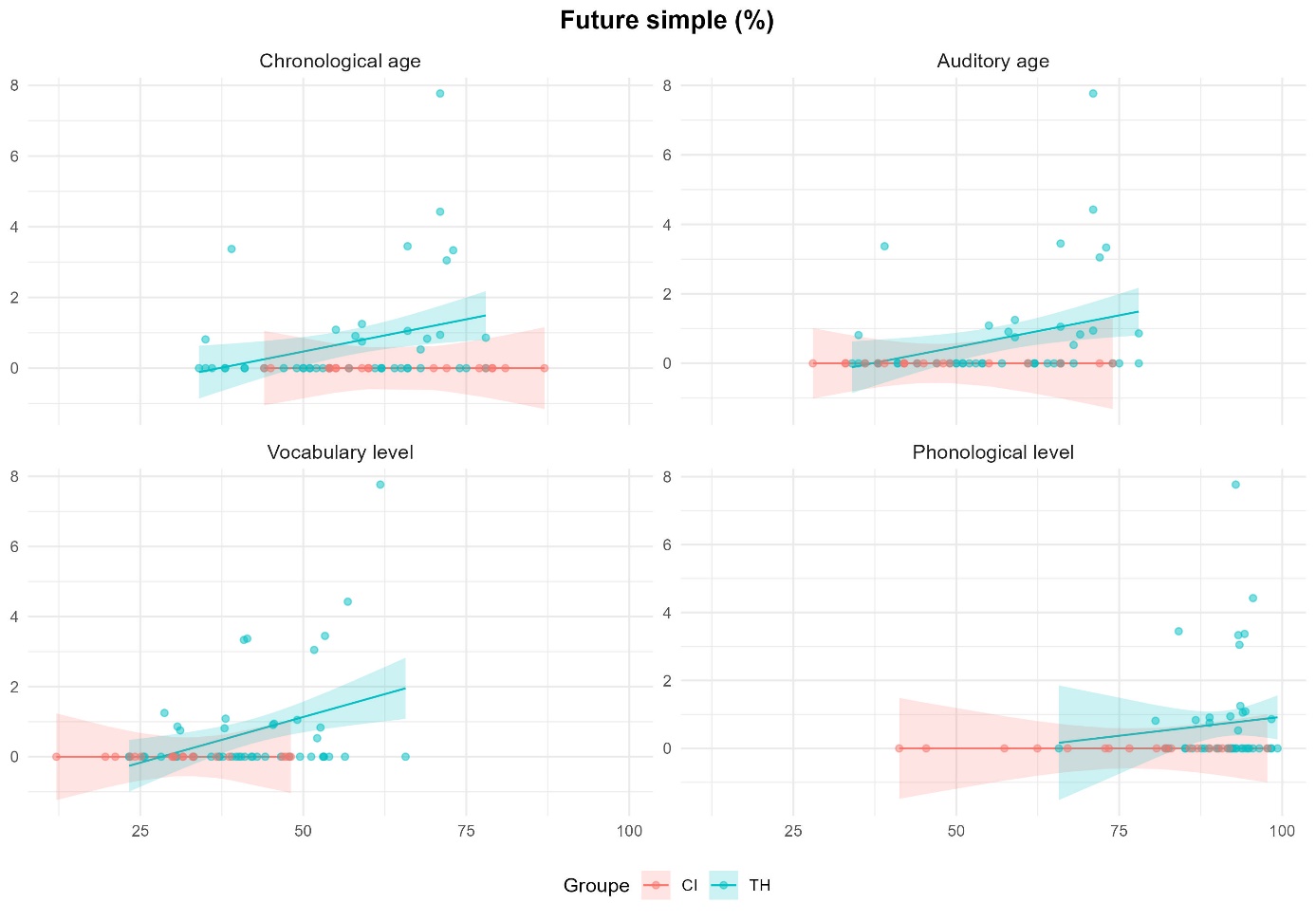


**Supplementary 2.2: Scatterplots of percentage scores percentages of future simples as a function of chronological age (top left), auditory age (top right) in months, vocabulary (bottom left), and phonological level (bottom right) for CI (red) and TH (blue) groups. Regression lines with 95% prediction intervals, based on the tested mixed models, are included.**


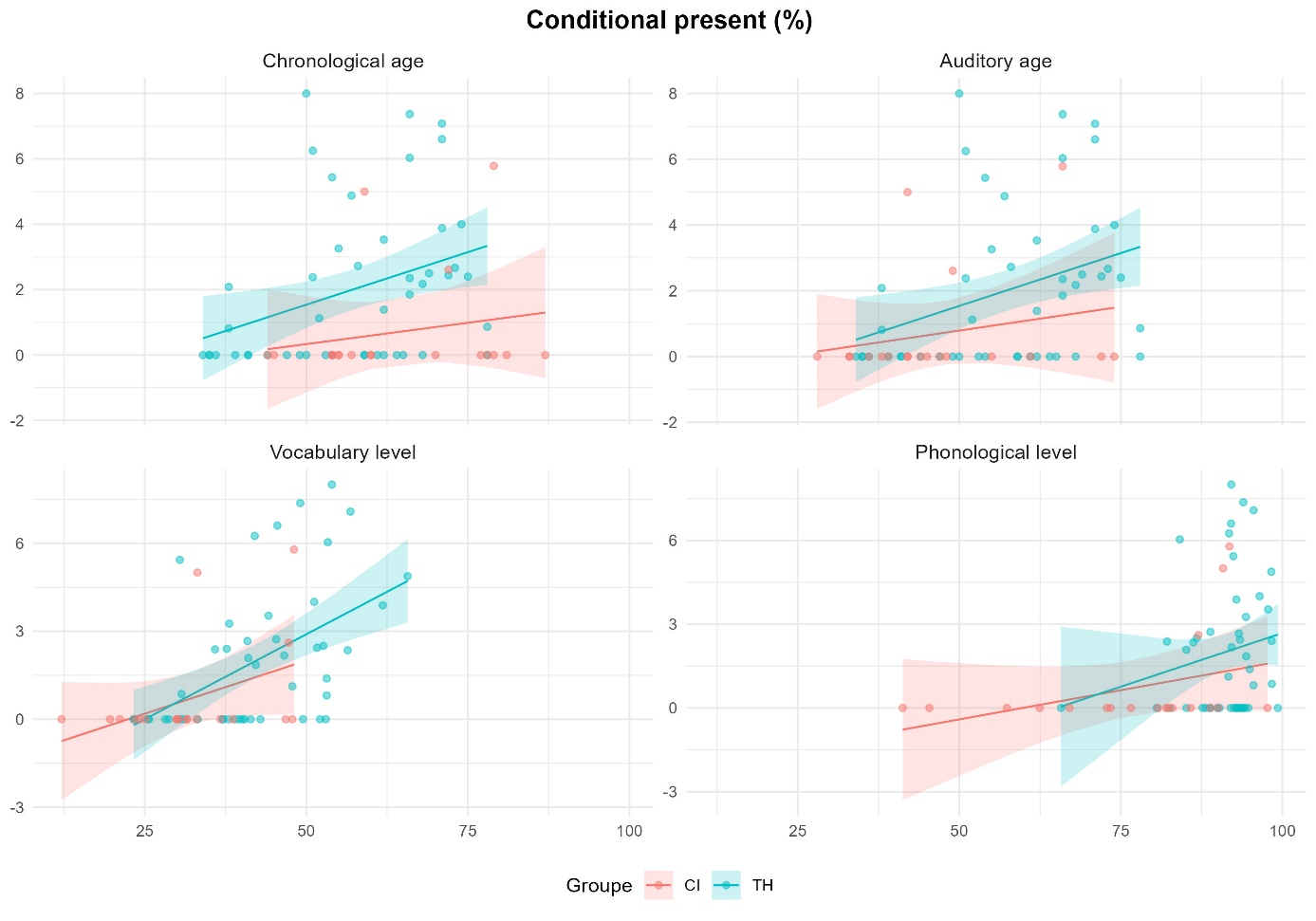
 **Supplementary 2.3: Scatterplots of percentage scores percentages of conditional presents as a function of chronological age (top left), auditory age (top right) in months, vocabulary (bottom left), and phonological level (bottom right) for CI (red) and TH (blue) groups. Regression lines with 95% prediction intervals, based on the tested mixed models, are included.**


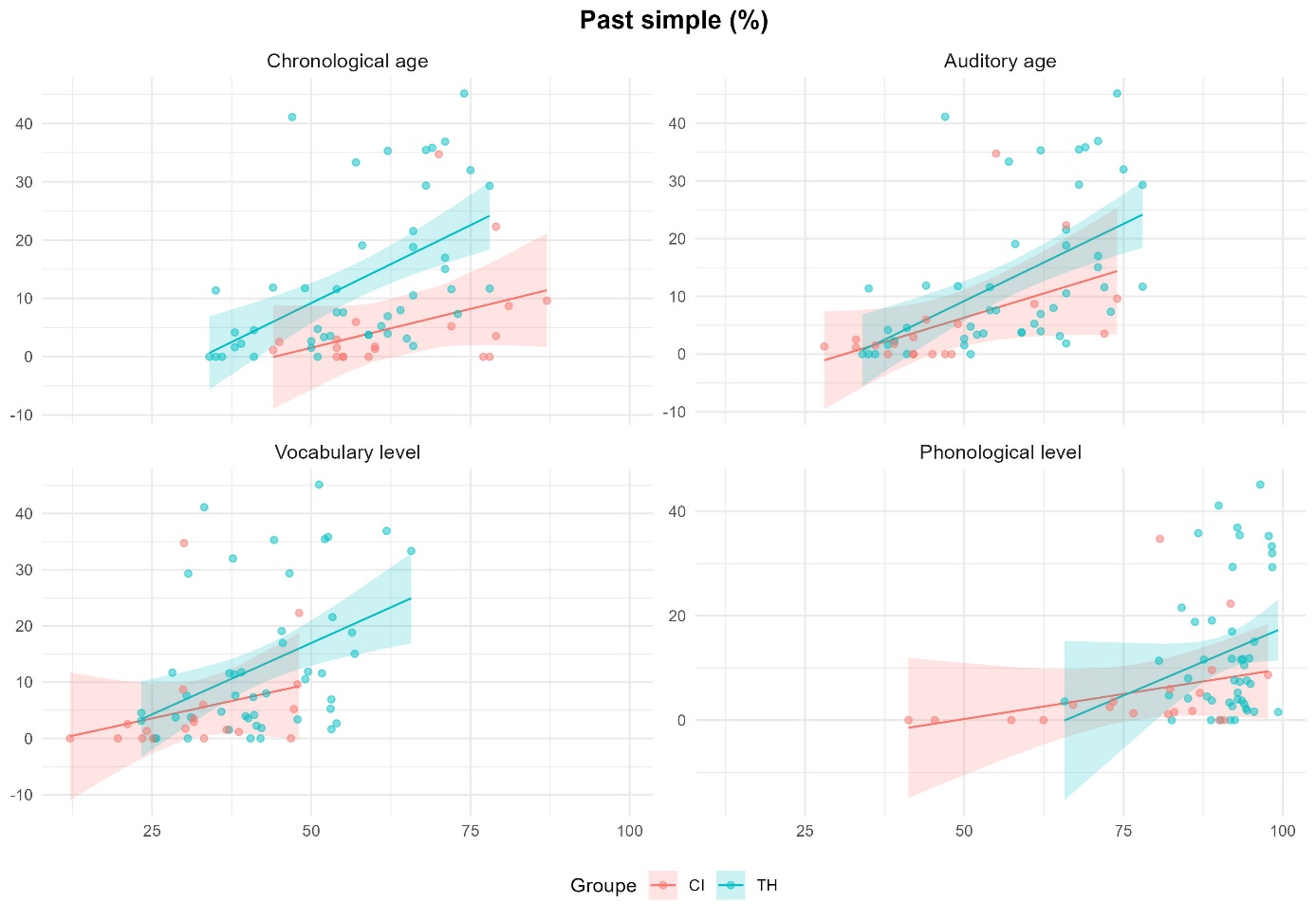
 **Supplementary 2.4: Scatterplots of percentage scores percentages of past simples as a function of chronological age (top left), auditory age (top right) in months, vocabulary (bottom left), and phonological level (bottom right) for CI (red) and TH (blue) groups. Regression lines with 95% prediction intervals, based on the tested mixed models, are included.**


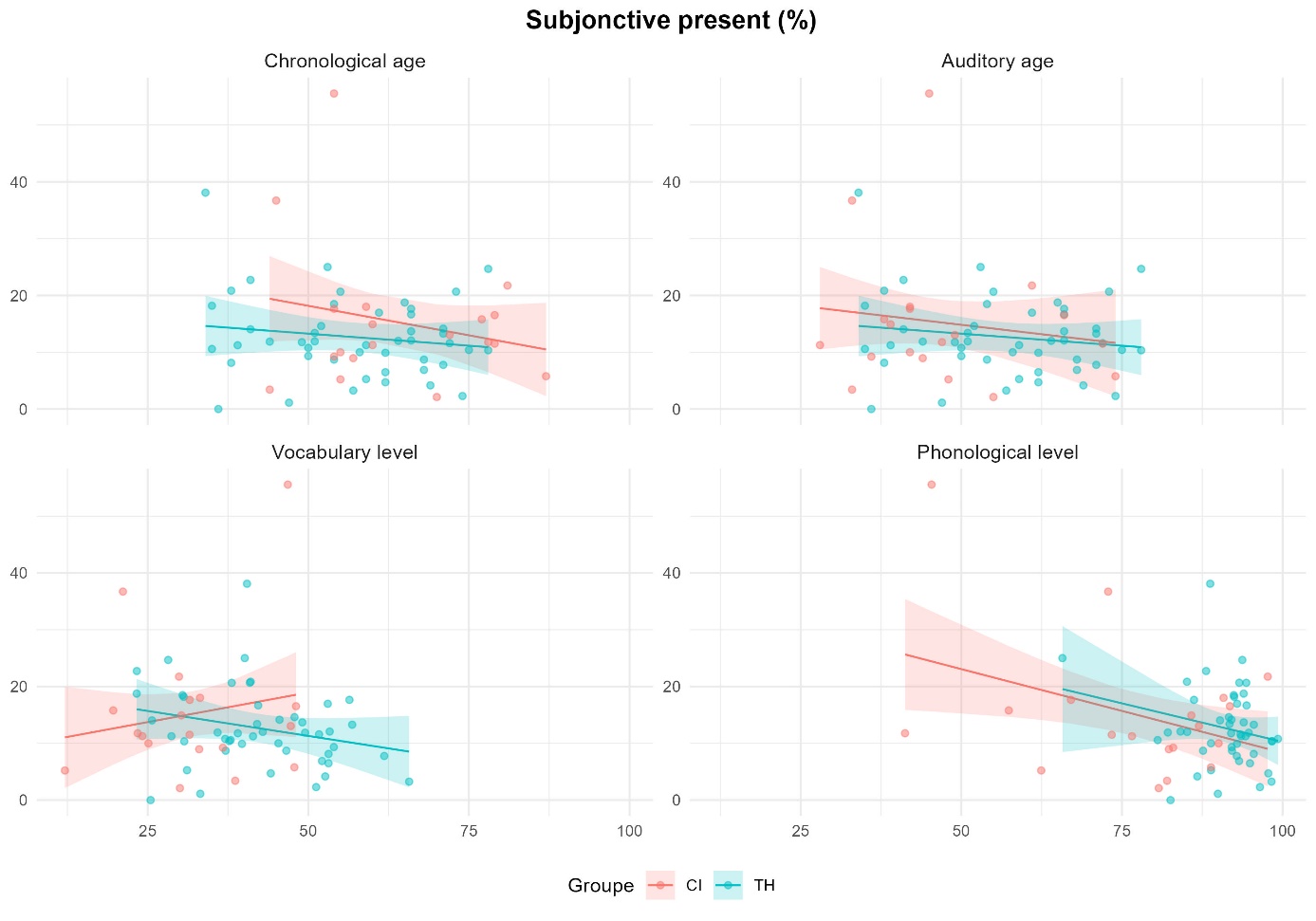
 **Supplementary 2.5: Scatterplots of percentage scores percentages of subjunctive presents as a function of chronological age (top left), auditory age (top right) in months, vocabulary (bottom left), and phonological level (bottom right) for CI (red) and TH (blue) groups. Regression lines with 95% prediction intervals, based on the tested mixed models, are included.**


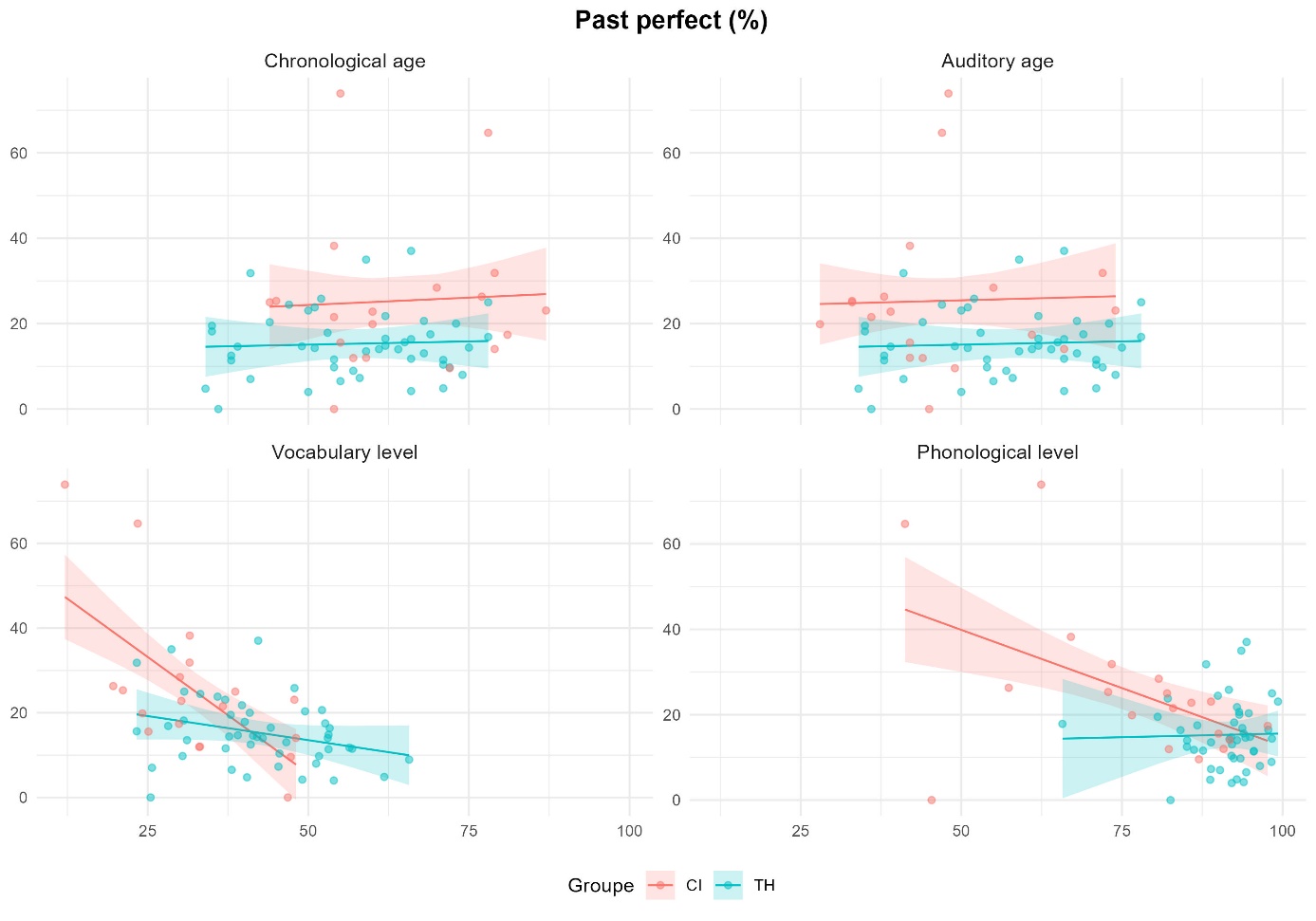


**Supplementary 2.6: Scatterplots of percentage scores percentages of past perfects as a function of chronological age (top left), auditory age (top right) in months, vocabulary (bottom left), and phonological level (bottom right) for CI (red) and TH (blue) groups. Regression lines with 95% prediction intervals, based on the tested mixed models, are included.**


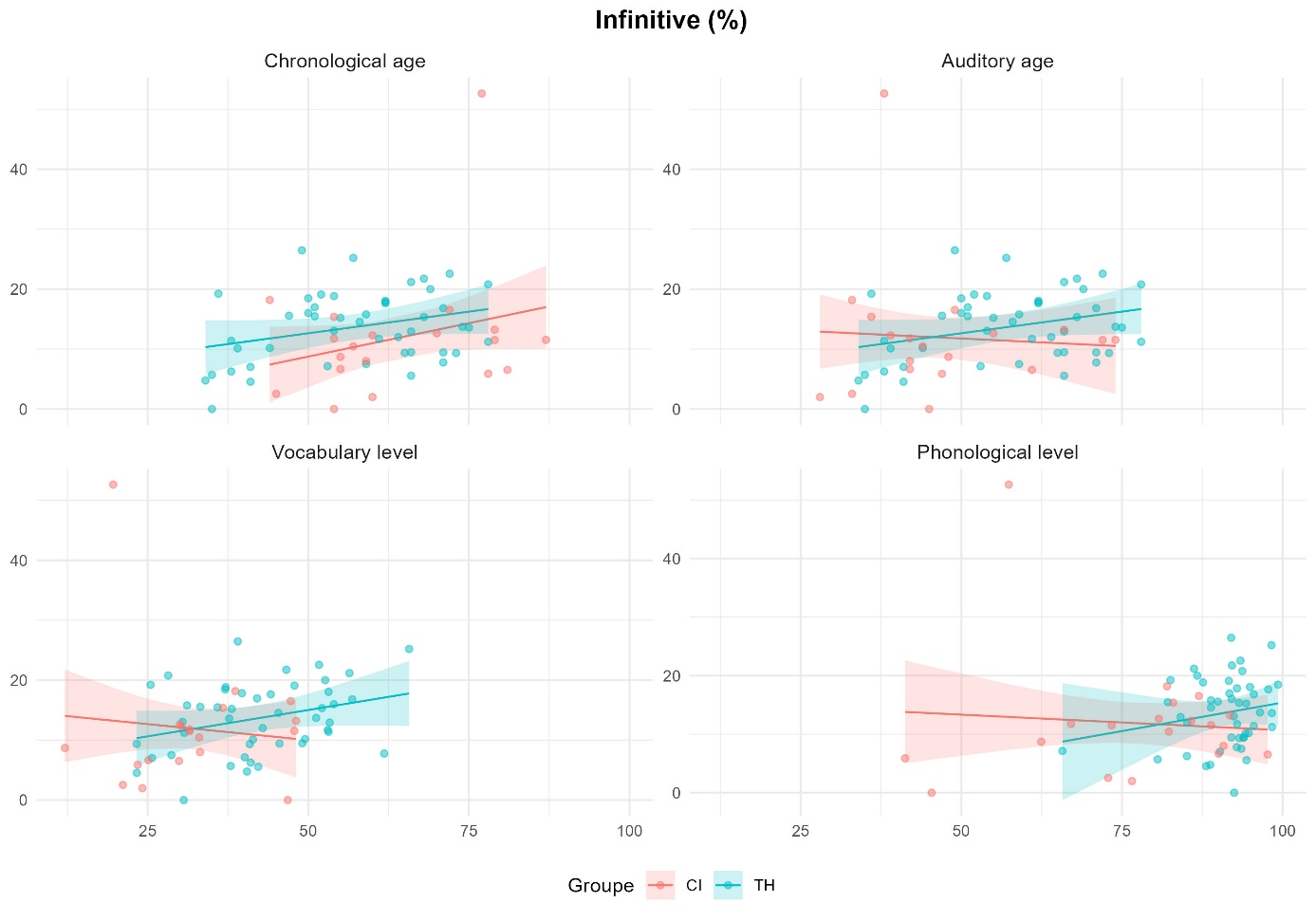


**Supplementary 2.7: Scatterplots of percentage scores percentages of infinitives as a function of chronological age (top left), auditory age (top right) in months, vocabulary (bottom left), and phonological level (bottom right) for CI (red) and TH (blue) groups. Regression lines with 95% prediction intervals, based on the tested mixed models, are included.**
