# Supplementary material for: Morphosyntactic production and processing skills in relation to age effects and lexical-phonological levels among children with cochlear implants and typically hearing peers: a focus on vowel nasality
Source: Front Hum Neurosci. 2025 Feb 26;19:1528388. doi: 10.3389/fnhum.2025.1528388 (PMC11897031; doi:10.3389/fnhum.2025.1528388)
Supplement: Supplementary file 3 [file Table_3.DOCX]

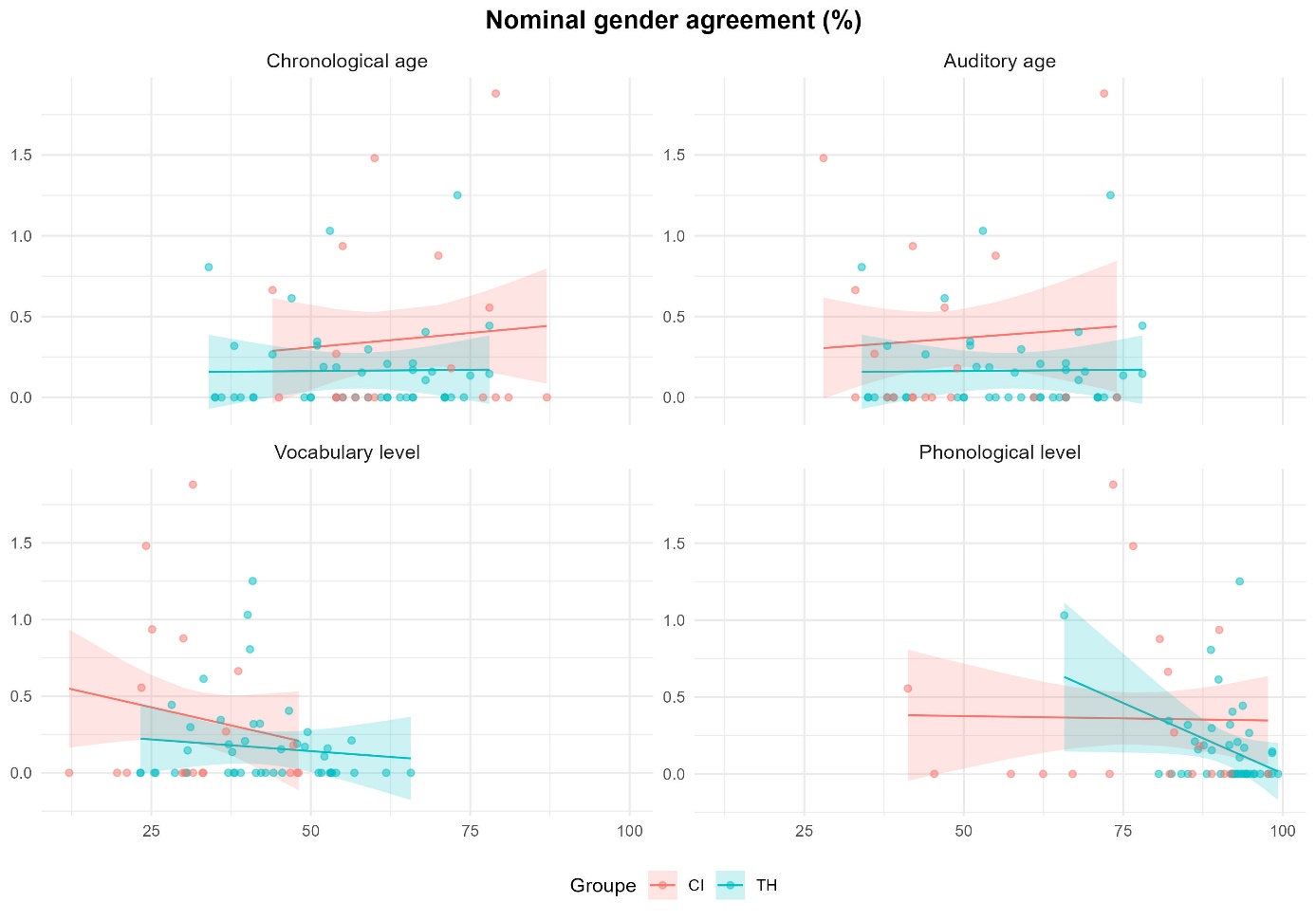


**Supplementary 3.1: Scatterplots of percentage scores percentages of nominal gender agreement errors as a function of chronological age (top left), auditory age (top right) in months, vocabulary (bottom left), and phonological level (bottom right) for CI (red) and TH (blue) groups. Regression lines with 95% prediction intervals, based on the tested mixed models, are included.**


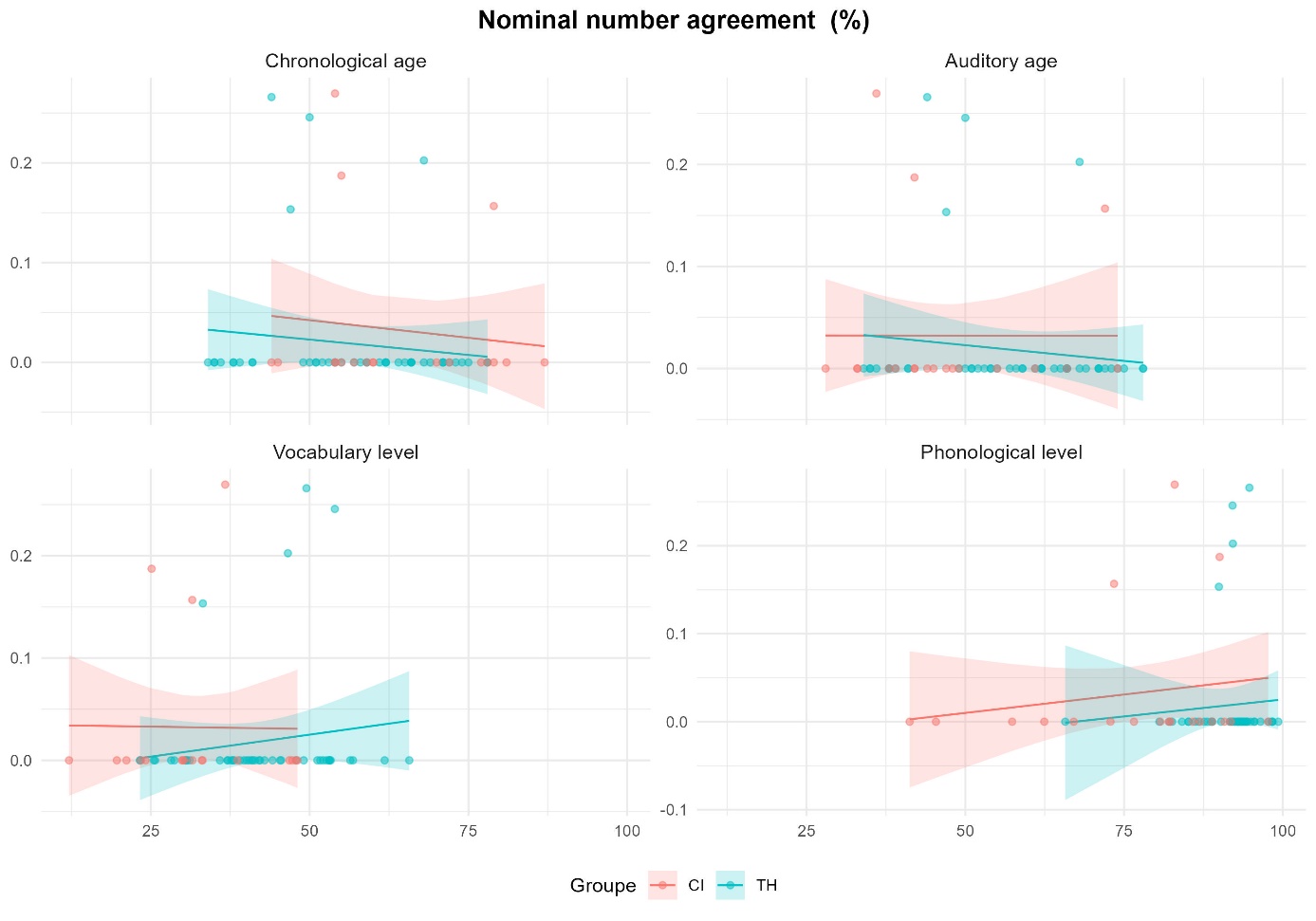


**Supplementary 3.2: Scatterplots of percentage scores percentages of nominal number agreement errors as a function of chronological age (top left), auditory age (top right) in months, vocabulary (bottom left), and phonological level (bottom right) for CI (red) and TH (blue) groups. Regression lines with 95% prediction intervals, based on the tested mixed models, are included.**


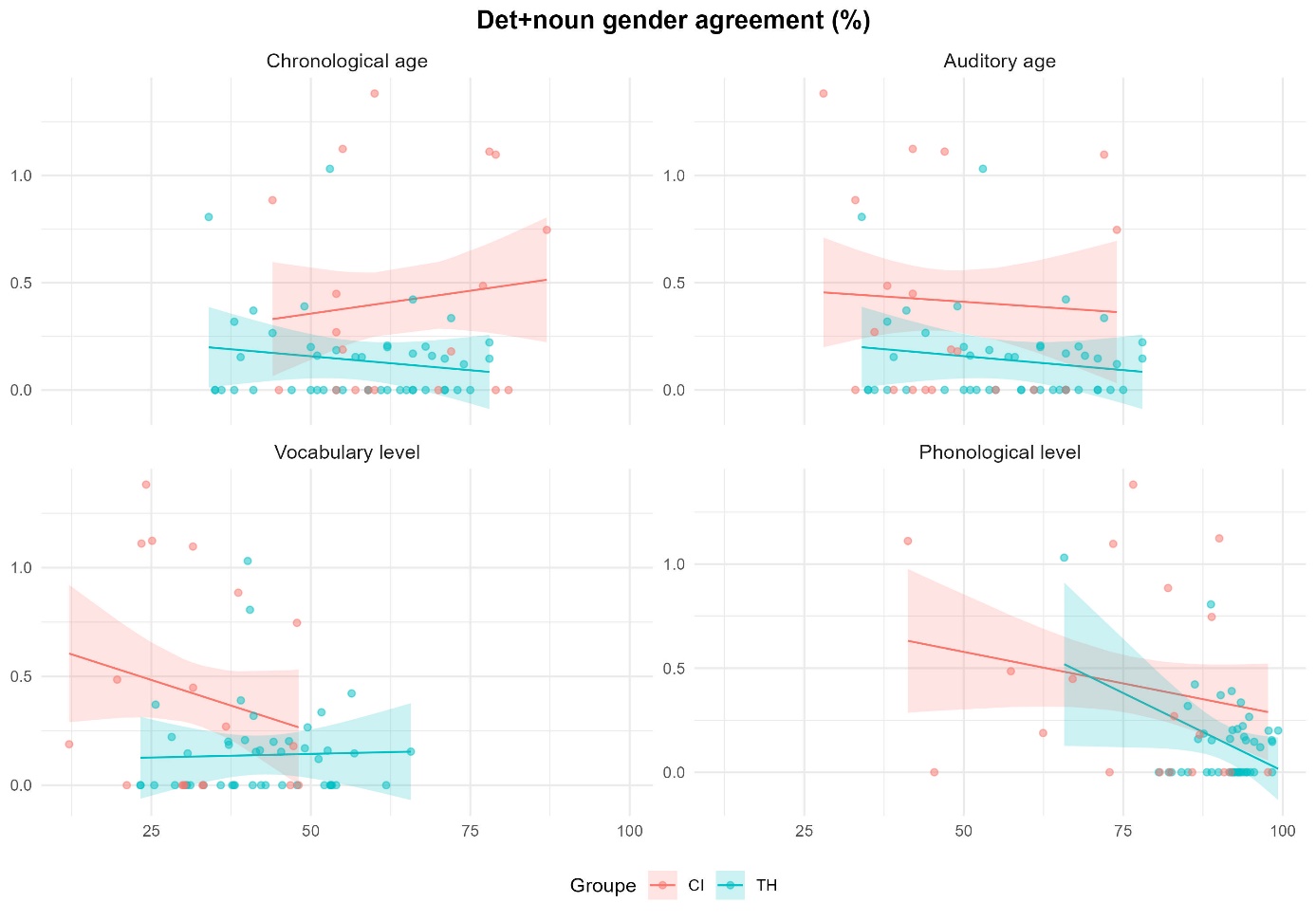


**Supplementary 3.3: Scatterplots of percentage scores percentages of the determiner+noun gender agreement errors as a function of chronological age (top left), auditory age (top right) in months, vocabulary (bottom left), and phonological level (bottom right) for CI (red) and TH (blue) groups. Regression lines with 95% prediction intervals, based on the tested mixed models, are included.**


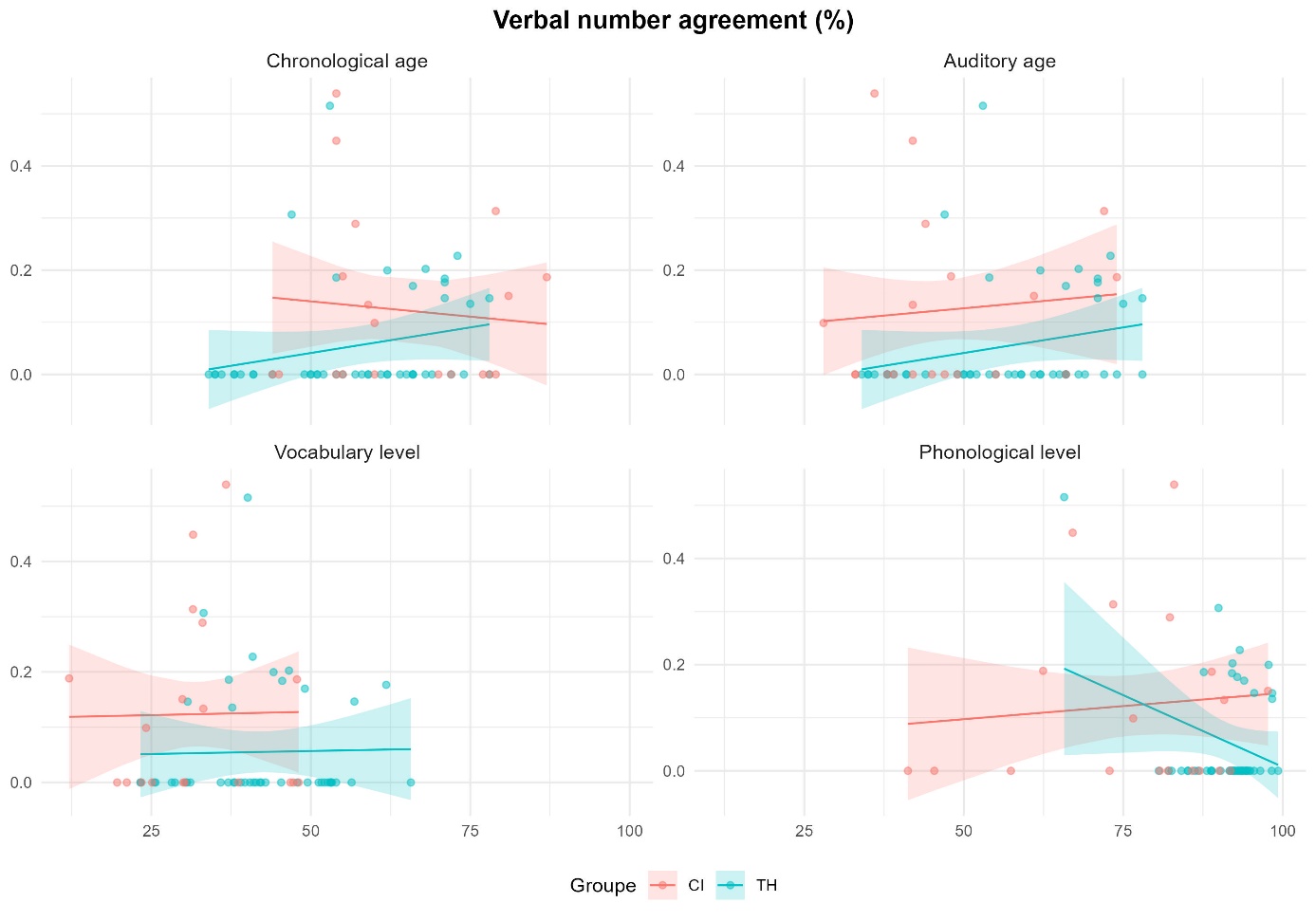


**Supplementary 3.4: Scatterplots of percentage scores percentages of verbal number agreement errors as a function of chronological age (top left), auditory age (top right) in months, vocabulary (bottom left), and phonological level (bottom right) for CI (red) and TH (blue) groups. Regression lines with 95% prediction intervals, based on the tested mixed models, are included.**


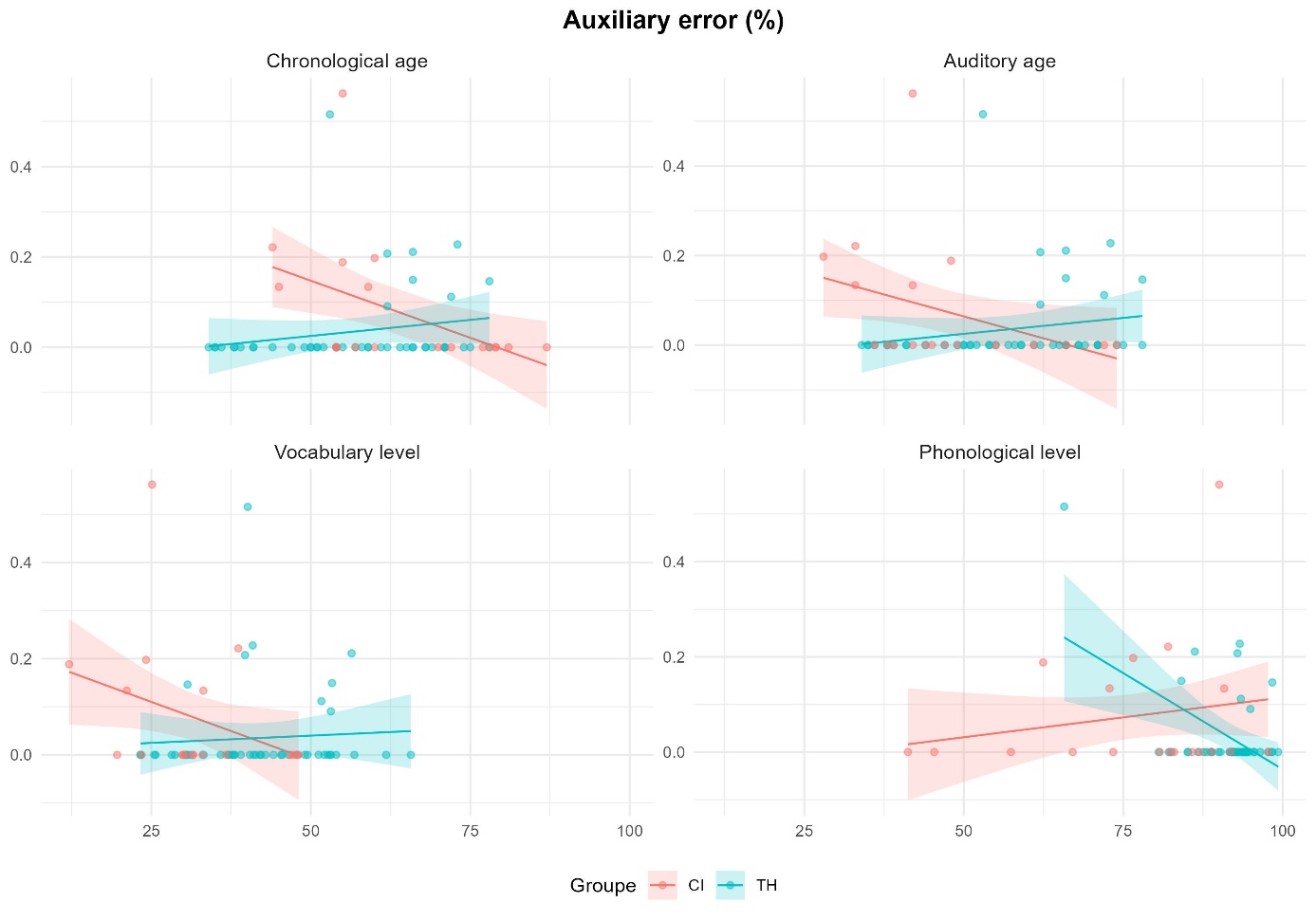


**Supplementary 3.5: Scatterplots of percentage scores percentages of auxiliary errors as a function of chronological age (top left), auditory age (top right) in months, vocabulary (bottom left), and phonological level (bottom right) for CI (red) and TH (blue) groups. Regression lines with 95% prediction intervals, based on the tested mixed models, are included.**


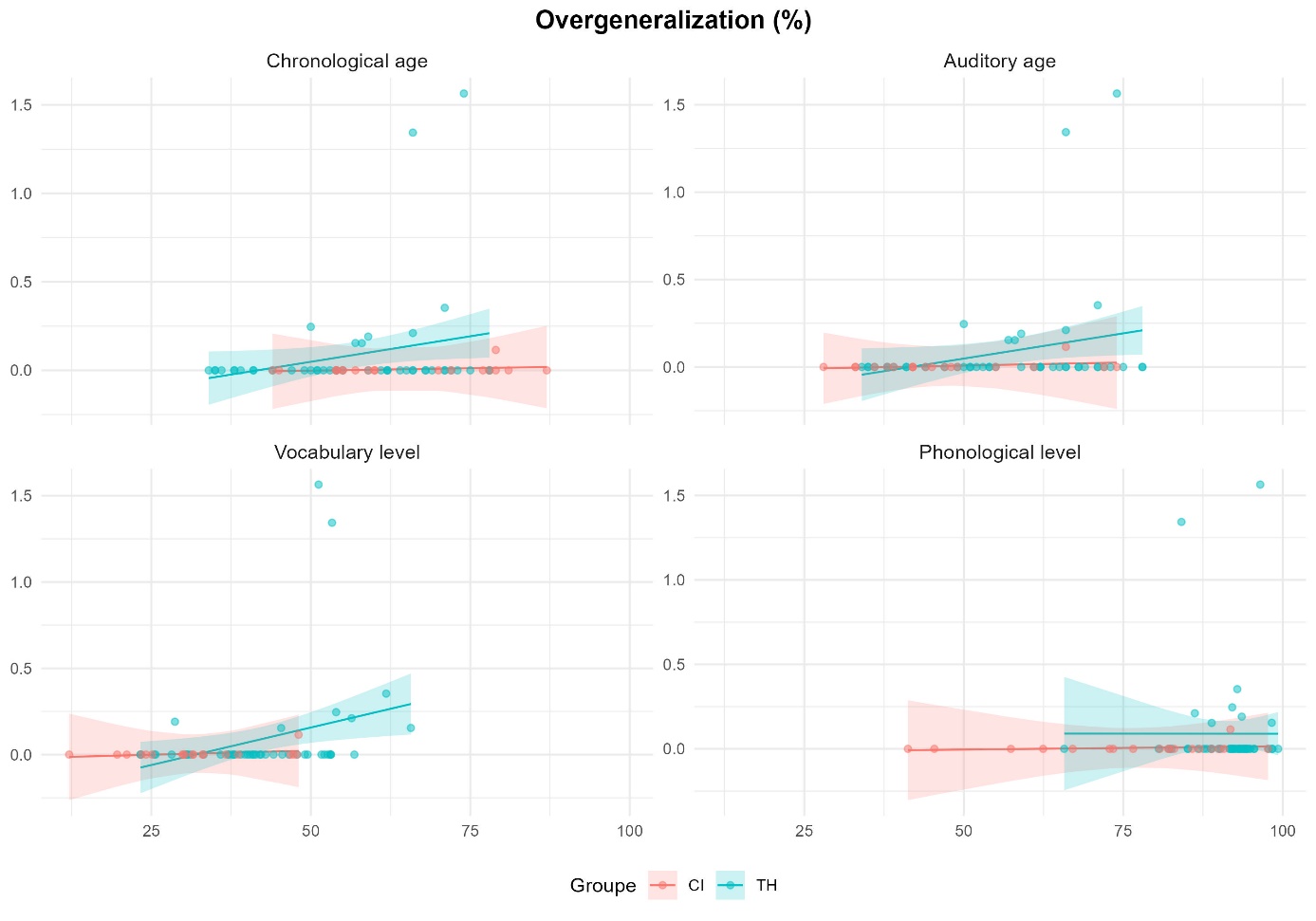


**Supplementary 3.6: Scatterplots of percentage scores percentages of overgeneralizations errors as a function of chronological age (top left), auditory age (top right) in months, vocabulary (bottom left), and phonological level (bottom right) for CI (red) and TH (blue) groups. Regression lines with 95% prediction intervals, based on the tested mixed models, are included.**


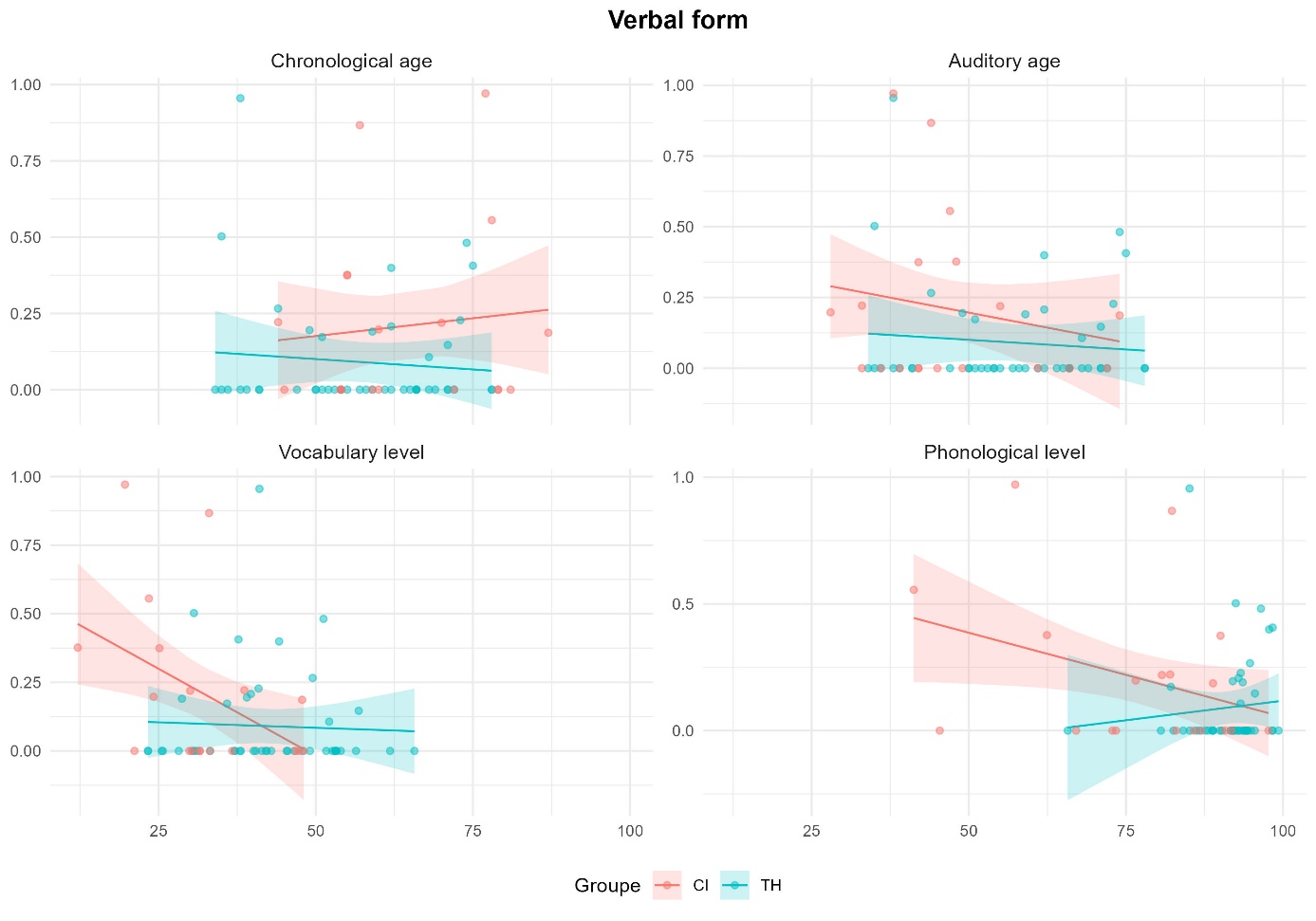


**Supplementary 3.7: Scatterplots of percentage scores percentages of verbal forms errors as a function of chronological age (top left), auditory age (top right) in months, vocabulary (bottom left), and phonological level (bottom right) for CI (red) and TH (blue) groups. Regression lines with 95% prediction intervals, based on the tested mixed models, are included.**


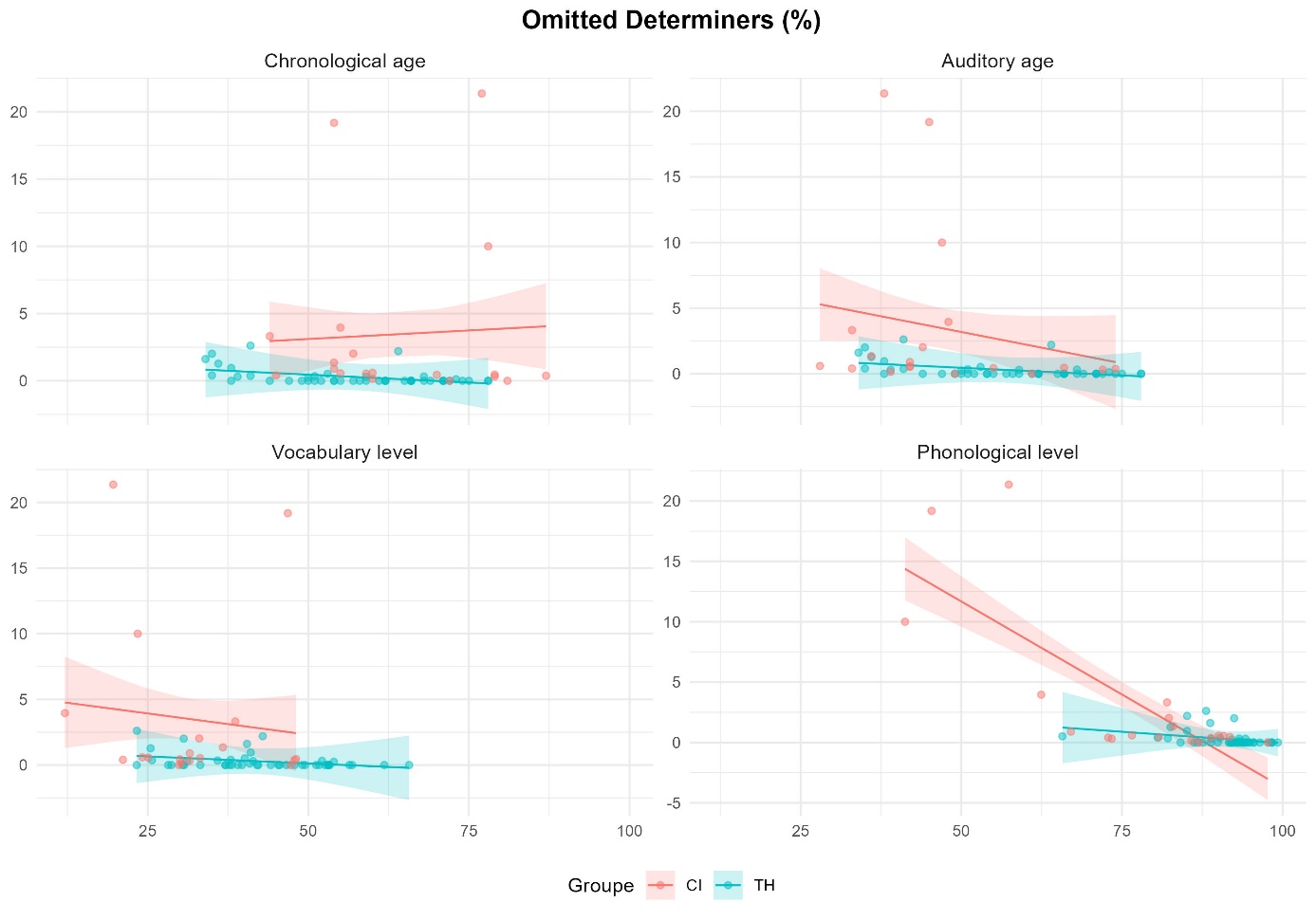


**Supplementary 3.8: Scatterplots of percentage scores percentages of omitted determiners as a function of chronological age (top left), auditory age (top right) in months, vocabulary (bottom left), and phonological level (bottom right) for CI (red) and TH (blue) groups. Regression lines with 95% prediction intervals, based on the tested mixed models, are included.**


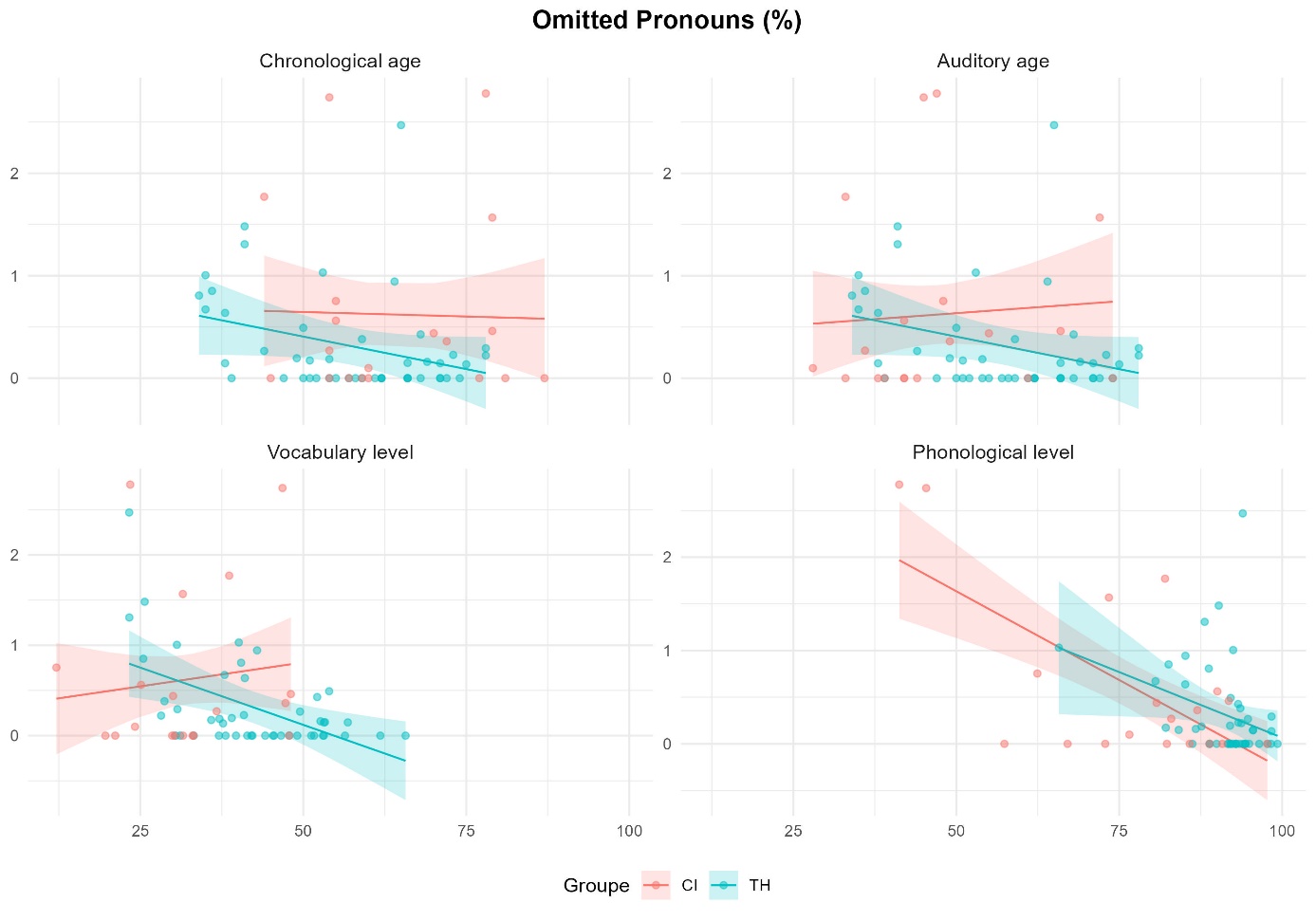
 **Supplementary 3.9: Scatterplots of percentage scores percentages of omitted pronouns as a function of chronological age (top left), auditory age (top right) in months, vocabulary (bottom left), and phonological level (bottom right) for CI (red) and TH (blue) groups. Regression lines with 95% prediction intervals, based on the tested mixed models, are included.**


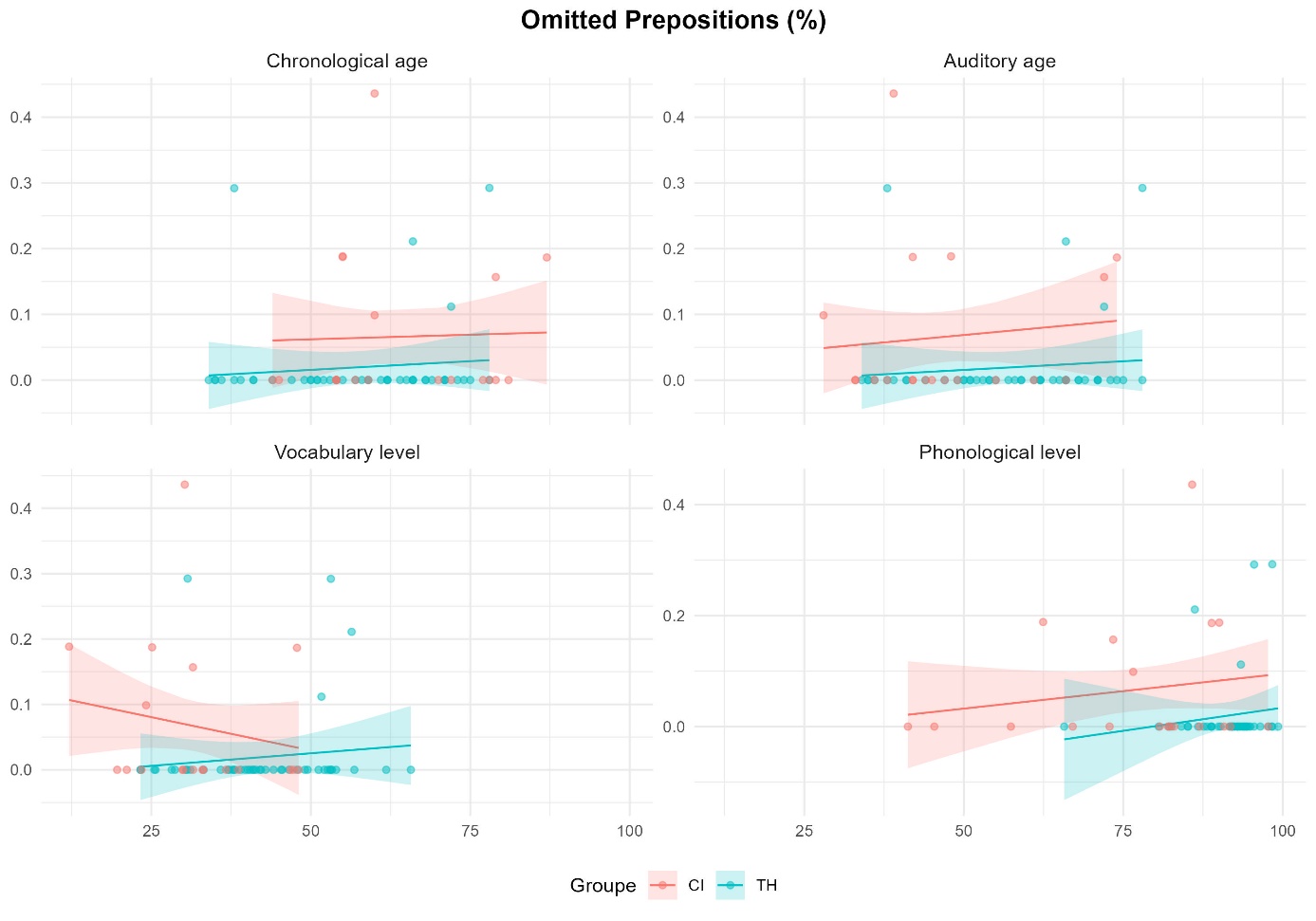


**Supplementary 3.10: Scatterplots of percentage scores percentages of omitted prepositions as a function of chronological age (top left), auditory age (top right) in months, vocabulary (bottom left), and phonological level (bottom right) for CI (red) and TH (blue) groups. Regression lines with 95% prediction intervals, based on the tested mixed models, are included.**


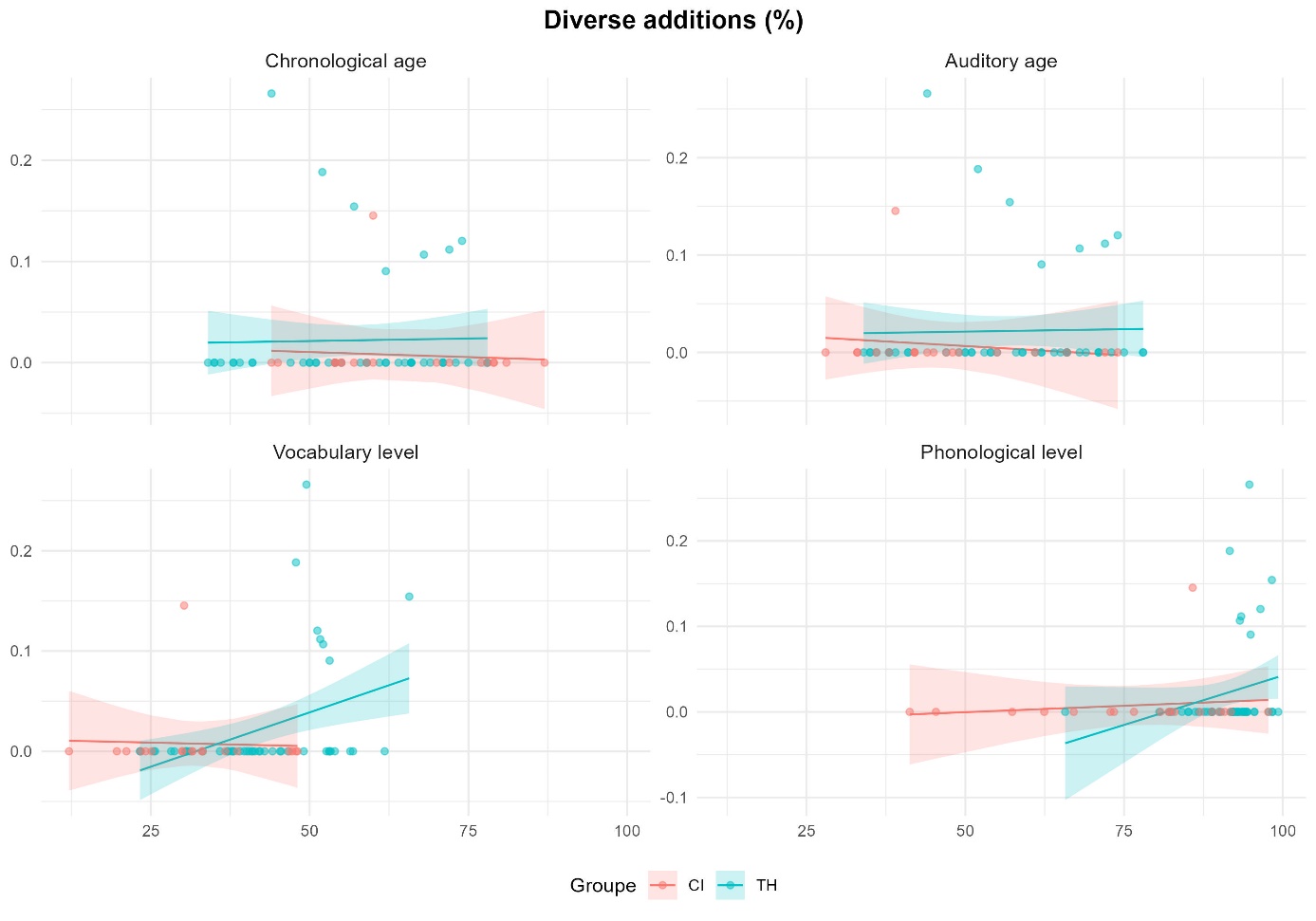


**Supplementary 3.11: Scatterplots of percentage scores percentages of diverse additions as a function of chronological age (top left), auditory age (top right) in months, vocabulary (bottom left), and phonological level (bottom right) for CI (red) and TH (blue) groups. Regression lines with 95% prediction intervals, based on the tested mixed models, are included.**
